# Tetrazole-containing naphthalene *bis*-sulfonamide Keap1-Nrf2 interaction inhibitors with unexpected binding modes

**DOI:** 10.1016/j.redox.2025.103924

**Published:** 2025-11-10

**Authors:** Nikolaos D. Georgakopoulos, Sandeep K. Talapatra, Sharadha Dayalan Naidu, Dina Dikovskaya, Maureen Higgins, Jemma Gatliff, Roxani Nikoloudaki, Marjolein Schaap, Jasmine M. Walker, Christopher Wardby, Albena T. Dinkova-Kostova, Sarah Harris, Frank Kozielski, Geoff Wells

**Affiliations:** a UCL School of Pharmacy, University College London, 29/39 Brunswick Square, London, WC1N 1AX, UK; b Jacqui Wood Cancer Centre, Division of Cellular Medicine, University of Dundee School of Medicine, Dundee, Scotland, DD1 9SY, UK; c Peninsula Medical School, University of Plymouth, Plymouth, Devon, PL4 8AA, UK; d Keregen Therapeutics Ltd, Stevenage Bioscience Catalyst, Gunnels Wood Rd, Stevenage, SG1 2FX, UK; e Department of Pharmacology and Molecular Sciences and Department of Medicine, Johns Hopkins University School of Medicine, Baltimore, MD, 21205, USA; f Department of Physics and Astronomy, Hicks Building, Hounsfield Rd, Broomhall, Sheffield, S3 7RH, UK

## Abstract

Naphthalene *bis*-sulfonamides are amongst the most studied inhibitors of the interaction between Kelch-like ECH-associated protein-1 (Keap1), a ubiquitination facilitator protein and the nuclear factor erythroid 2-related factor 2 (Nrf2), a transcription factor associated with diverse cytoprotective responses. The compounds bind to the C-terminal Kelch domain of Keap1 and occupy the Nrf2 binding pocket, causing Nrf2 to accumulate and increase the expression of a battery of antioxidant response element genes. The binding modes of the compounds have been characterised through a series of X-ray crystallography and binding assay studies. In this work we have evaluated a series of symmetric and asymmetric naphthalene *bis*-sulfonamides and identified a tight binding acid/tetrazole derivative **25**. We have determined its distinct binding mode to Keap1 using X-ray crystallography, biophysical binding assays and its Nrf2 activation using cell-based assays. We have also characterised its conformational interconversions in solution using NMR. In contrast to previous studies, we have shown that the compound can bind to Keap1 in both its ‘cis’ and ‘trans’ rotational states that are both occupied and interconvert in solution. Molecular dynamics simulations indicate that the *cis*-form has the lowest interaction energy, but the *trans*-form is more stable in solution. The compound binds tightly to Keap1 (low nanomolar *K*_d_), indicating that the non-standard binding modes may contribute significantly to the high affinity. The study highlights the need for caution when interpreting the structure-activity relationships of closely related compounds in this series and will inform further work on these compounds.

## Highlights

• Asymmetric naphthalene *bis*-sulphonamides such as 25 bind tightly to Keap1.
• The compounds have non-standard binding mode revealed by X-ray crystallography.
• Compound 25 induces the expression of Nrf2 target genes in cells.
• The study informs the further development of this important class of Nrf2 inducer.

## Graphical abstract

**Figure:**
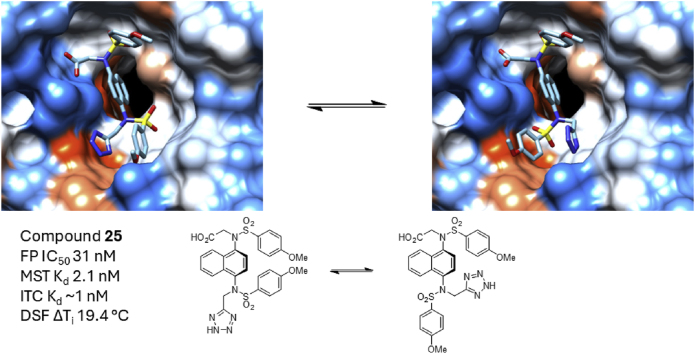

## 1 Introduction

Inhibiting the interaction between Kelch-like ECH-associated protein 1 (Keap1) and nuclear factor erythroid 2-related factor 2 (Nrf2) has been a recent focus of drug discovery efforts in academia and the pharmaceutical industry. Nrf2 is a bZip transcription factor that interacts with antioxidant response elements (AREs) in the promoter region of a battery of genes that regulate the activity of proteins involved in xenobiotic metabolism, autophagy, mitochondrial function and antioxidant activities. Increasing Nrf2 activity has been proposed as a therapeutic intervention for a range of conditions spanning neurodegenerative diseases such as Parkinson's and Alzheimer's diseases, inflammatory conditions, and chronic obstructive pulmonary disease [1]. Two electrophilic Keap1 binders have been approved for clinical use, omaveloxolone for Friedreich's ataxia [2] and dimethyl fumarate for relapsing forms of multiple sclerosis [3]. These compounds interact with redox-sensitive cysteine residues in Keap1 to inhibit Nrf2 ubiquitination [4,5]. In contrast, targeting the Keap1-Nrf2 protein-protein interaction (PPI) has the potential to enhance the activity of Nrf2 without covalent modification of Keap1 or other proteins, it has been proposed that this may improve the selectivity and pharmaceutical properties of the inhibitors [1,6].

**Fig. 1:**
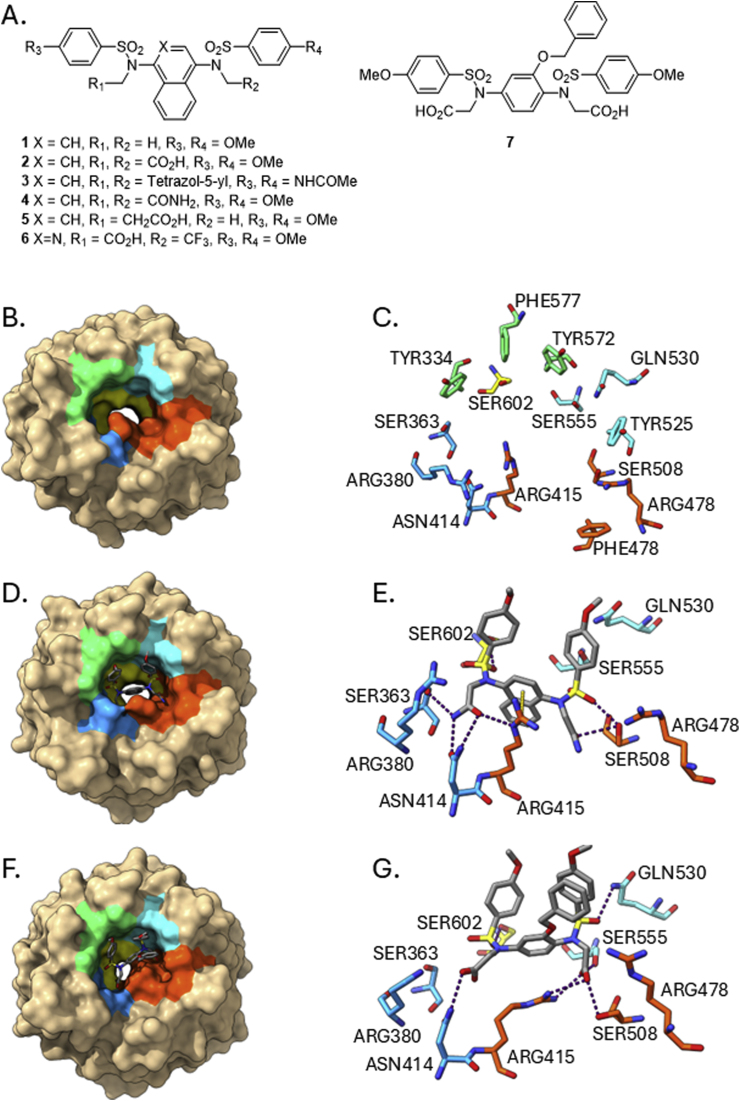
A. Representative *bis*-sulfonamide inhibitors of the Keap1-Nrf2 protein-protein interaction; B. The Keap1 Kelch domain (residues 322–609) shown in surface representation; sub-pockets of the Nrf2 binding site are P1 (orange, residues 415, 461, 462, 478, 483 & 508), P2 (blue, 363, 380, 381 & 414), P3 (yellow, 364, 509, 556, 571, 602 & 603), P4 (green, 334, 572 & 577) and P5 (cyan, 525, 530 & 555); C. stick representation of binding site residues; Equivalent representations of the binding modes of selected compounds: D. & E. Binding mode of compound **4**; F. & G. Binding mode of compound **7** in the Keap1 Kelch domain. Images were created using UCSF ChimeraX [9]. Hydrogen bonds were assigned using the ChimeraX H-Bonds tool and the default parameters and are shown as purple dotted lines. Pi-cation interactions (Fig. 1E, shown as a yellow line) were identified where the arginine guanidine and small molecule aromatic moieties were stacked and separated by a distance of ∼4 Å [10,11], a line was created between the centre of each group using markers in ChimeraX.

Several classes of small molecule Keap1-Nrf2 PPI inhibitors have been identified and highlighted in recent reviews [7,8]. The inhibitors bind within the C-terminal Kelch repeat-containing domain (residues 322–609) of Keap1 and occupy the Nrf2 binding pocket. Amongst the chemotypes of Keap1 inhibitor that have been developed (Fig. 1A), the aryl *bis*-sulfonamide group of structures have been extensively evaluated and characterised in terms of their structure activity relationships and *in vitro* cellular activities on Nrf2 target gene expression [[12], [13], [14], [15], [16], [17], [18], [19], [20], [21], [22], [23], [24]]. Most of the compounds have at least one carboxylic acid moiety (or carboxylate equivalent) that interacts with one or more arginine residues in the binding pocket and a central aromatic ring that lies in the P3 subpocket that forms a water-filled channel through the centre of the domain (apo protein and binding site, Fig. 1B and C).

The *bis*-sulfonamide compounds have two principal binding modes, the most prevalent involved the central bicyclic moiety, usually a naphthalene or an isoquinoline, occupying the central P3 subpocket, the two aryl sulfonamide groups occupying the P4 and P5 subpockets and the polar N-alkyl substituents in the P1 and P2 subpockets [18]. In this binding orientation ARG415 between subpockets 1 and 2 is rotated to form potential pi-cation interactions with the bicyclic aromatic moiety (Fig. 1D and E). Recently we have also characterised a new closed-pocket conformation of the binding pocket for *bis*-sulfonamides with a central monocyclic phenyl ring. In this form of the protein the central subpocket is partially occluded by ARG415 and the substituents occupy the remaining four subpockets. For these structures intramolecular pi-stacking interactions are particularly important to the bound conformation of the compound (Fig. 1F and G) [23].

In this work we have compared and characterised a series of symmetrical and asymmetrically substituted naphthalene *bis*-sulfonamides for their ability to bind to Keap1. The studies revealed that the conventional understanding of the SAR for this series is further complicated by alternative binding modes via rotation around the naphthalene-sulphonamide bonds. We have evaluated this behaviour by characterising the compounds’ rotamers using NMR measurements and their binding behaviour using X-ray crystallography, Keap1 binding assays and molecular dynamics simulations. The cellular activity of the compounds is comparable to other polar naphthalene sulfonamide compounds, but the binding mode information informs the further development of compounds in this series for use as either non-electrophilic Nrf2 inducers or E3 ligase binders for proteolysis targeting chimeras (PROTACs) in a range of therapeutic applications.

## 2 Materials and methods

### 2.1 General

All anhydrous solvents and reagents were purchased from commercial suppliers and used without further purification.

^1^H NMR spectra were recorded at ambient temperature in deuterated solvents (CDCl_3_ or DMSO-d_6_) on a Bruker Advance 400 Spectrophotometer at 400.13 MHz or Bruker Advance 500 Spectrophotometer at 500 MHz. Chemical shifts are reported in parts per million (ppm) downfield from the tetramethylsilane reference (*δ* = 0) using the residual protonated solvent as an internal standard (^1^H: *δ* (CDCl_3_) = 7.26 ppm, *δ* (DMSO-*d6*) = 2.50 ppm). Data for ^1^H NMR is given as follows: chemical shift (multiplicity, coupling constants (J, given in Hertz (Hz)), integration and assignment). Multiplicities in the ^1^H NMR spectra are quoted as: s = singlet, d = doublet, t = triplet, q = quartet, m = multiplet, dd = double doublet, ddd = double double doublet. Splitting patterns that could not be interpreted or easily visualised were recorded as multiplets (m) or broad peaks (br).

^13^C NMR spectra were recorded at ambient temperature in deuterated solvents (CDCl_3_ or DMSO-d_6_) on a Bruker Advance 400 Spectrophotometer at 100.61 MHz, or Bruker Advance 500 Spectrophotometer at 125 MHz. Chemical shifts were measured in ppm relative to tetramethylsilane (*δ* = 0) using the following internal references: *δ* (CDCl_3_) = 77.0 ppm, *δ* (DMSO-*d6*) = 39.4 ppm.

High-resolution mass spectrometry (HRMS) spectra were recorded on a Micromass Q-TOF Premier Tandem Mass Spectrometer coupled to an HPLC instrument using electrospray ionisation (ESI) mass spectrometry. Calibration was performed with an internal standard – positive mode: reserpine which gives *m*/*z* [M+H]^+^ = 609.2812, negative mode: taurocholic acid which gives *m*/*z* [M − H]^-^ = 514.2839.

Analytical TLC was performed on pre-coated Merck glass backed silica gel plates (Silica gel 60 F_254_) and visualised by exposure to ultraviolet light (254 nm) and/or staining with an appropriate reagent followed by heating if required.

Flash column chromatography was carried out on Merck Kieselgel 60 (40–63 μm) under a positive pressure of N_2_ gas.

LC-MS spectra were recorded using a Shimadzu LCMS-2020 equipped with an XTerra® MS C18 column (4.6 × 50 mm, 2.5 μm) and a flow rate of 0.6 mL/min. The eluent system consisted of eluent A (H_2_O with 0.1 % formic acid, HPLC grade) and eluent B (MeCN with 0.1 % formic acid, HPLC grade) with the following conditions: 0.0–2.5 min 90 % A: 10 % B, then a linear gradient from 2.5 min to 5.5 min to a final composition of 5 % A: 95 % B that was maintained for a further 2.5 min, then adjusted to 10 % A: 90 % B over 10.5 min and held for 1.5 min.

Analytical reverse-phase HPLC was carried out on an XSELECT™ CSH™ C18 column 50 × 6 mm (particle size: 2.5 μm) at a flow rate of 1.0 mL/min. The eluent system consisted of eluent A (H_2_O with 0.1 % TFA, HPLC grade) and eluent B (MeCN with 0.1 % TFA, HPLC grade) with the following gradient conditions: initial fixed composition 5 % B to 95 % B over 15 min, held for 2 min at 95 % B.

### 2.2 General methods

#### 2.2.1 Synthesis of symmetrical *bis*-sulfonamides (Method A)

A mixture of the nitro compound (1 eq.) and SnCl_2_·2H_2_O (5 eq.) in EtOH (7 mL/mmol) was stirred at 65 °C for 2 h. On completion, the reaction mixture was diluted with EtOAc, basified to pH 8–9 with sat. NaHCO_3_ and the resulting tin salts were removed by filtration. The organic layer was isolated and the aqueous layer was further extracted with EtOAc. The combined organic portions were washed with water, dried over anhydrous Na_2_SO_4_ and evaporated to dryness. The crude amine was used without further purification in the next step.

To a solution of the crude *bis*-amine (1 eq.) and the sulfonyl chloride (2.2 eq.) in DCM (4 mL/mmol) at room temperature (rt) were added pyridine (3 eq.) and a catalytic amount of DMAP (0.1 eq.) and the reaction mixture was stirred overnight. Following the addition of a small volume of hexane to aid precipitation, the crude solid was collected by filtration and crystallised from an appropriate solvent to yield the pure product.

#### 2.2.2 Alkylation of *bis*-sulfonamides (Method B)

To a solution of the *bis*-sulfonamide (1 eq.) in *N*,*N*-dimethylformamide (DMF) (2 mL/mmol) were added K_2_CO_3_ (3 eq.) and the halo-substituted electrophile (2.5 eq.) and the reaction mixture was stirred at rt for 4 h. The reaction was quenched with H_2_O and acidified with 1n HCl to pH 5. The resulting precipitate was collected by filtration, washed with H_2_O and dried in a vacuum desiccator overnight.

#### 2.2.3 Ethyl ester deprotection (Method C)

To a solution of the ethyl ester (1 eq.) in THF/MeOH (1:1 v/v) was added 1n NaOH (5 eq.) and the reaction was stirred at rt for 2 h. On completion, the reaction was acidified with 1n HCl to pH 1–2 and the resulting precipitate was collected by filtration, washed thoroughly with H_2_O and dried in a vacuum desiccator overnight.

#### 2.3 Compound Synthesis

##### 2.3.1 1-Nitronapthalene (9)

To a 250 mL flask equipped with a reflux condenser and CaCl_2_ drying tube was added naphthalene **8** (12 g, 93.63 mmol), KNO_3_ (9.5 g, 93.67 mmol), TFAA (46 mL, 327.71 mmol) and CHCl_3_ (94 mL) and the reaction was stirred at rt for 5 h. On completion, the reaction mixture was poured into H_2_O (200 mL) and extracted with CHCl_3_ (3 x 80 mL). The organic layer was washed with H_2_O (3 x 100 mL), dried over anhydrous MgSO_4_ and evaporated to dryness to afford 1-nitronapthalene **9** (14.7 g, 91 %) as a yellow solid. ^1^H NMR (500 MHz, CDCl_3_) *δ* (ppm): 8.59 (d, *J* = 8.8 Hz, 1H), 8.26 (d, *J* = 7.6 Hz, 1H), 8.14 (d, *J* = 8.2 Hz, 1H), 7.98 (d, *J* = 8.2 Hz, 1H), 7.74 (t, *J* = 7.7 Hz, 1H), 7.65 (t, *J* = 7.5 Hz, 1H), 7.57 (t, *J* = 7.9 Hz, 1H). ^13^C NMR (125 MHz, CDCl_3_) *δ* (ppm): 146.5, 134.8, 134.4, 129.5, 128.7, 127.4, 125.2, 124.2, 124.1, 123.2. The above data are in agreement with those previously reported in the literature [25,26].

##### 2.3.2 4-Nitronaphthalen-1-amine (10)

The title compound was prepared using an adaptation of a published procedure (Jiang et al., 2014)[13]. To a suspension of 1-nitronaphthalane **9** (10.0 g, 57.75 mmol) and NH_2_OH·HCl (25.0 g, 35.97 mmol) in EtOH (700 mL) at 60 °C, was added dropwise over a period of 1 h a filtered solution of KOH (50.0 g, 89.11 mmol) in MeOH (250 mL). The reaction mixture was stirred for 1 h and was then poured, while warm, into ice-cold water (2 L). The resulting precipitate was collected by filtration, washed thoroughly with H_2_O and crystallised from MeCN to yield the amine **10** (7.9 g, 73 %) as off-white crystals. ^1^H NMR (400 MHz, DMSO-*d*_6_) *δ* (ppm): 8.90 (d, *J* = 8.8 Hz, 1H), 8.39 (d, *J* = 8.9 Hz, 1H), 8.30 (d, *J* = 8.4 Hz, 1H), 7.82–7.69 (m, 1H), 7.59–7.51 (m, 3H), 6.69 (d, *J* = 9.0 Hz, 1H). ^13^C NMR (100 MHz, DMSO-*d*_6_) *δ* (ppm): 153.5, 131.6, 130.3, 127.9, 124.9, 123.5, 123.3, 120.1, 105.3. The above data are in agreement with those previously reported in the literature [27].

##### 2.3.3 *N*,*N*'-(Naphthalene-1,4-diyl)*bis*(4-methoxybenzenesulfonamide) (1)

Prepared from compound **10** (3.0 g, 15.94 mmol) and 4-methoxybenzenesulfonyl chloride (6.9 g, 33.48 mmol) according to the general method A. Crystallisation from MeCN afforded **1** (3.9 g, 49 %) as white crystals. ^1^H NMR (400 MHz, DMSO-*d*_6_) *δ* (ppm): 10.01 (s, 2H), 7.96 (dd, *J* = 6.5, 3.3 Hz, 2H), 7.59–7.52 (m, 4H), 7.39 (dd, *J* = 6.5, 3.3 Hz, 2H), 7.03–6.95 (m, 6H), 3.78 (s, 6H). ^13^C NMR (100 MHz, DMSO-*d*_6_) *δ* (ppm): 162.3, 131.4, 131.0, 130.0, 128.8, 126.0, 123.3, 122.6, 114.8, 55.8. LC-MS: *m/z* (ESI) = 498.65 [M−H]^−^, t_R_ = 6.05 min, purity: >95 %. The above data are in agreement with those previously reported in the literature [13].

##### 2.3.4 Diethyl 2,2'-(naphthalene-1,4-diyl*bis*(((4-methoxyphenyl)sulfonyl) azanediyl))diacetate (11)

Prepared from **1** (1.0 g, 20.04 mmol) and ethyl bromoacetate (550 μL, 5.01 mmol) according to the general method B. Crystallisation from EtOAc/hexane afforded **11** (1.0 g, 74 %) as white crystals. ^1^H NMR (500 MHz, DMSO-*d*_6_) *δ* (ppm): 8.31 (dd, *J* = 6.5, 3.3 Hz, 1H), 8.18 (dd, *J* = 6.4, 3.2 Hz, 1H), 7.64–7.55 (m, 6H), 7.16–7.04 (m, 5H), 6.85 (m, 1H), 4.53–4.43 (m, 4H), 4.08–3.95 (m, 4H), 3.87 (s, 3H), 3.85 (s, 3H), 1.08–1.01 (m, 6H). ^13^C NMR (125 MHz, DMSO-*d*_6_) *δ* (ppm): 168.5, 168.6, 162.9, 162.5, 137.7, 137.0, 133.0, 132.1, 130.5, 129.1, 129.0, 128.7, 126.0, 126.7, 126.6, 126.5, 124.7, 124.4, 114.6, 60.6, 60.0, 59.5, 55.7, 55.5, 53.3, 53.1, 13.9, 13.2. The above data are in agreement with those previously reported in the literature [13].

##### 2.3.5 2,2'-(Naphthalene-1,4-diyl*bis*(((4-methoxyphenyl)sulfonyl)azanediyl))diacetic acid (2)

Prepared from **11** (800 g, 1.19 mmol) according to the general method C. The crude product was triturated with DCM to afford **2** (478 mg, 65 %) as a white powder. ^1^H NMR (500 MHz, DMSO-*d*_6_) *δ* (ppm): 13.00–12.64 (br, 2H), 8.30 (dd, *J* = 6.4, 3.2 Hz, 1H), 8.17 (dd, *J* = 6.4, 3.2 Hz, 1H), 7.61–7.53 (m, 6H), 7.16–7.01 (m, 5H), 6.88 (s, 1H), 4.40 (ddd, *J* = 31.0, 17.8, 12.7 Hz, 4H), 3.88 (s, 3H), 3.83 (s, 3H). ^13^C NMR (125 MHz, DMSO-*d*_6_) *δ* (ppm): 169.9, 169.8, 162.9, 162.8, 137.1, 137.0, 132.9, 132.3, 130.2, 129.8, 129.4, 128.8, 126.9, 126.6, 126.3, 126.1, 124.8, 124.4, 114.8, 114.4, 55.5, 55.3, 53.2, 53.1. HRMS (ESI): calculated for C_28_H_26_N_2_O_10_S_2_ [M+H]^+^ 615.1107, found 615.1102. HPLC retention time: t_R_ = 8.78 min, purity: >95 %. The above data are in agreement with those previously reported in the literature [13].

##### 2.3.6 2,2'-(Naphthalene-1,4-diyl*bis*(((4-methoxyphenyl)sulfonyl)azanediyl))diacetamide (4)

To a solution of **2** (55 mg, 0.09 mmol) in dry DMF (1 mL) were added successively dry pyridine (14 μL, 0.18 mmol), Boc_2_O (58 mg, 0.27 mmol), and (NH_4_)_2_CO_3_ (13 mg, 0.13 mmol) and the reaction mixture was stirred under Ar for 19 h at rt. On completion, H_2_O (10 mL) was added and the resulting precipitate was collected by filtration, washed with H_2_O and Et_2_O and dried *in vacuo* to give **4** (42 mg, 77 %) as a white powder. ^1^H NMR (500 MHz, DMSO-*d*_6_) *δ* (ppm): 8.29 (dd, *J* = 6.5, 3.3 Hz, 1H), 8.19 (dd, *J* = 6.4, 3.2 Hz, 1H), 7.61–7.41 (m, 6H), 7.35–7.31 (br, 1H), 7.30–7.25 (br, 1H), 7.18–6.81 (m, 8H), 4.35–4.11 (m, 4H), 3.89 (s, 3H), 3.84 (s, 3H). ^13^C NMR (125 MHz, DMSO-*d*_6_) *δ* (ppm): 168.7, 162.8, 162.7, 136.9, 133.0, 132.9, 130.2, 130.0, 129.5, 129.1, 126.5, 126.3, 126.0, 124.7, 114.2, 114.1, 55.7, 55.3, 54.0, 53.9. HRMS (ESI): calculated for C_28_H_28_N_4_O_8_S_2_ [M−H]^−^ 611.1270, found 611.1250. HPLC retention time: t_R_ = 7.48 min, purity: >95 %. The above data are in agreement with those previously reported in the literature [18].

##### 2.3.7 *N*,*N*'-(Naphthalene-1,4-diyl)*bis*(*N*-(cyanomethyl)-4-methoxybenzenesulfonamide) (12)

Prepared from **1** (200 mg, 0.40 mmol) and bromoacetonitrile (70 μL, 1.00 mmol) according to the general method B. Crystallisation from toluene afforded **12** (148 mg, 67 %) as off-white crystals. ^1^H NMR (400 MHz, DMSO-*d*_6_) *δ* (ppm): 8.18–8.00 (m, 2H), 7.87–7.59 (m, 6H), 7.28–6.87 (m, 6H), 5.06–4.88 (m, 4H), 3.91 (s, 3H), 3.87 (s, 3H). ^13^C NMR (100 MHz, DMSO-*d*_6_) *δ* (ppm): 163.4, 136.8, 136.3, 132.9, 130.2, 130.0, 128.6, 128.0, 127.9, 126.3, 126.0, 123.6, 116.2, 114.7, 55.8. HRMS (ESI): calculated for C_28_H_24_N_4_O_6_S_2_ [M+H]^+^ 599.1035, found 599.1035. HPLC retention time: t_R_ = 10.31 min, purity: >95 %.

##### 2.3.8 *N*,*N*'-(Naphthalene-1,4-diyl)*bis*(*N*-((1*H*-tetrazol-5-yl)methyl)-4-methoxybenzenesulfonamide) (13)

To a solution of **12** (50 mg, 0.09 mmol) in DMF (2 mL), was added NaN_3_ (30 mg, 0.44 mmol) and NH_4_Cl (30 mg, 0.56 mmol) and the reaction was stirred at 100 °C for 16 h. On completion, the reaction mixture was diluted with H_2_O (20 mL), extracted with EtOAc (3 x 20 mL) and the combined organic layers were washed with H_2_O (3 x 80 mL), sat. brine (3 x 80 mL), dried over anhydrous MgSO_4_ and evaporated to dryness under reduced pressure. The crude product was purified by flash chromatography (DCM/EtOAc/formic acid 70:29:1 v/v) to afford **13** (18 mg, 31 %) as a white solid. ^1^H NMR (400 MHz, DMSO-*d*_6_) *δ* (ppm): 8.13 (dd, *J* = 6.5, 3.3 Hz, 1H), 8.04 (dd, *J* = 6.5, 3.3 Hz, 1H), 7.65–7.46 (m, 6H), 7.17 (d, *J* = 8.9 Hz, 2H), 7.08 (d, *J* = 8.9 Hz, 2H), 6.82–6.69 (m, 2H), 5.34–5.18 (m, 2H), 5.13–4.95 (m, 2H), 3.91 (s, 3H), 3.86 (s, 3H). ^13^C NMR (100 MHz, DMSO-*d*_6_) *δ* (ppm): 163.1, 163.0, 136.5, 135.4, 133.6, 132.5, 130.2, 130.1, 130.0, 129.9, 128.7, 128.3, 126.6, 126.4, 126.2, 125.2, 124.0, 123.5, 114.5, 114.4, 114.1, 55.7, 55.4, 45.4, 44.8. HRMS (ESI): calculated for C_28_H_26_N_10_O_6_S_2_ [M−H]^−^ 661.1400, found 661.1373. HPLC retention time: t_R_ = 8.37 min, purity: >95 %.

##### 2.3.9 Acetohydrazide (14)

The title compound was prepared using an adaptation of a published procedure [28]. To a solution of EtOAc (10 mL, 102.38 mmol) in EtOH (40 mL) was added hydrazine hydrate (5.0 mL, 102.38 mmol). The reaction mixture was stirred under reflux for 24 h, followed by removal of EtOH *in vacuo* to afford the acetohydrazide **14** (6.1 g, 81 %) as a white solid. ^1^H NMR (500 MHz, DMSO-*d*_6_) *δ* (ppm): 8.92 (s, 1H), 4.13 (s, 2H), 1.74 (s, 3H). ^13^C NMR (125 MHz, DMSO-*d*_6_) *δ* (ppm): 168.6, 20.5.

##### 2.3.10 *N*'-Acetyl-2-chloroacetohydrazide (15)

The title compound was prepared using an adaptation of a published procedure [29]. To a solution of acetohydrazide **14** (4.0 g, 51.28 mmol) and Na_2_CO_3_ (3.2 g, 30.33 mmol) in H_2_O (20 mL) at 0 °C was added dropwise over a period of 20 min chloroacetyl chloride (4.3 mL, 53.84 mmol) and the reaction mixture was allowed to warm to rt and was stirred for 1 h. The precipitate was collected by filtration and dried *in vacuo* to afford the compound **15** (9.0 g, 45 %) as white powder. ^1^H NMR (500 MHz, DMSO-*d*_6_) *δ* (ppm): 10.19 (s, 1H), 9.97 (s, 1H), 4.11 (s, 2H), 1.87 (s, 3H). ^13^C NMR (125 MHz, DMSO-*d*_6_) *δ* (ppm): 167.9, 164.8, 40.3, 20.4. The above data are in agreement with those previously reported in the literature [30].

##### 2.3.11 *N*,*N*'-(Naphthalene-1,4-diyl)*bis*(4-methoxy-*N*-((5-methyl-1,3,4-oxadiazol-2-yl)methyl)benzenesulfonamide) (17)

To a solution of *N*′-acetyl-2-chloroacetohydrazide **15** (2.0 g, 12.99 mmol) in anhydrous acetonitrile (5 mL) at 0 °C under Ar, POCl_3_ (1.2 mL, 12.99 mmol) was added dropwise over a period of 10 min and the resulting mixture was heated to 80 °C and stirred for 3 h. After the reaction was cooled to rt, H_2_O (50 mL) was added and the mixture was extracted with DCM (3 x 50 mL). The combined organic layers were washed with H_2_O (3 x 100 mL), sat. NaHCO_3_ (3 x 100 mL) and sat. brine (3 x 100 mL), dried over anhydrous MgSO_4_ and evaporated under reduced pressure to afford the crude 2-chloromethyl-5-methyl-1,3,4-oxadiazole **16** as a dark yellow oil, which was used in the next step without further purification.

The crude oxadizaole **16** (500 mg, 4.21 mmol) was reacted with **1** (700 mg, 1.40 mmol) according to the general method C. Purification by flash chromatography (EtOAc 100 %) afforded **16** (450 mg, 47 %) as a white powder. ^1^H NMR (400 MHz, DMSO-*d*_6_) *δ* (ppm): 8.08 (dd, *J* = 6.5, 3.3 Hz, 1H), 8.00 (dd, *J* = 6.5, 3.3 Hz, 1H), 7.68–7.53 (m, 6H), 7.19 (d, *J* = 9.0 Hz, 2H), 7.10 (d, *J* = 9.0 Hz, 2H), 6.97 (s, 1H), 6.82 (s, 1H), 5.30–4.88 (m, 4H), 3.91 (s, 3H), 3.86 (s, 3H), 2.40 (s, 3H), 2.36 (s, 3H). ^13^C NMR (100 MHz, DMSO-*d*_6_) *δ* (ppm): 164.9, 163.1, 163.0, 162.9, 162.2, 136.8, 136.4, 133.0, 132.8, 130.1, 129.0, 128.8, 128.6, 127.1, 126.6, 126.2, 123.6, 114.4, 55.7, 55.2, 46.3, 46.0, 10.8, 10.4. LC-MS: *m/z* (ESI) = 1381.25 [2 M + H]^+^, t_R_ = 5.78 min, >95 %. HRMS (ESI): calculated for C_27_H_26_N_2_O_7_S_2_ [M−H]^−^ 691.1645, found 691.1641. HPLC retention time: t_R_ = 9.32 min, purity: >95 %.

##### 2.3.12 4-Methoxy-*N*-(4-nitronaphthalen-1-yl)benzenesulfonamide (18)

To a solution of 4-nitro-1-naphthylamine **10** (4.6 g, 24.44 mmol) in toluene/pyridine (50 mL, 1:1 v/v) was added 4-methoxybenzenesulfonyl chloride (5.7 g, 27.58 mmol) and the resulting suspension was heated to 100 °C for 2 h. On completion, the solvent was evaporated *in vacuo* and the resulting residue was diluted with 1n HCl (100 mL) and extracted with EtOAc (3 x 100 mL). The combined organic layers were washed with 1n HCl (3 x 100 mL), H_2_O (3 x 100 mL) and sat. brine (3 x 100 mL), dried over anhydrous Na_2_SO_4_ and evaporated to dryness under reduced pressure. The crude product was triturated with Et_2_O, EtOH and crystallised from toluene to afford the nitro compound **18** (5.9 g, 68 %) as off-white crystals. ^1^H NMR (400 MHz, DMSO-*d*_6_) *δ* (ppm): 10.81 (s, 1H), 8.39 (d, *J* = 8.4 Hz, 1H), 8.28 (t, *J* = 8.0 Hz, 2H), 7.81–7.71 (m, 3H), 7.67 (ddd, *J* = 8.2, 6.9, 1.1 Hz, 1H), 7.43 (d, *J* = 8.5 Hz, 1H), 7.09–6.99 (m, 2H), 3.78 (s, 3H). ^13^C NMR (125 MHz, DMSO-*d*_6_) *δ* (ppm): 162.6, 142.8, 138.9, 130.9, 129.7, 128.9, 127.6, 127.3, 125.2, 124.7, 123.7, 122.6, 117.8, 114.5, 55.6. The above data are in agreement with those previously reported in the literature [18].

##### 2.3.13 Ethyl 2-(4-methoxy-*N*-(4-nitronaphthalen-1-yl)phenylsulfonamido) acetate (19)

To a solution of **18** (5.8 g, 16.06 mmol) in DMF (3 mL) was added ethyl bromoacetate (2.3 mL, 20.07 mmol) and K_2_CO_3_ (3.3 g, 23.95 mmol) and the reaction was stirred for 24 h at rt. On completion, H_2_O (30 mL) was added to the reaction and the resulting precipitate was collected by filtration, washed with H_2_O and Et_2_O and dried *in vacuo*. The crude product was purified by flash chromatography (DCM/hexane 6:4 v/v) to afford the ester **19** (3.2 g, 45 %) as an off-white fluffy powder. ^1^H NMR (400 MHz, DMSO-*d*_6_) *δ* (ppm): 8.41–8.25 (m, 3H), 7.83 (ddd, *J* = 8.5, 6.9, 1.3 Hz, 1H), 7.75 (ddd, *J* = 8.2, 6.9, 1.2 Hz, 1H), 7.68–7.60 (m, 2H), 7.36 (d, *J* = 8.2 Hz, 1H), 7.15–7.09 (m, 2H), 4.58 (s, 2H), 4.03 (q, *J* = 7.1 Hz, 2H), 3.86 (s, 3H), 1.08 (t, *J* = 7.1 Hz, 3H). ^13^C NMR (100 MHz, DMSO-*d*_6_) *δ* (ppm): 168.2, 163.1, 146.2, 141.4, 132.3, 130.0, 129.7, 128.6, 127.1, 126.3, 125.4, 125.1, 123.1, 122.10, 114.5, 60.4, 55.7, 52.9, 13.7. The above data are in agreement with those previously reported in the literature [18].

##### 2.3.14 Ethyl 2-(4-methoxy-*N*-(4-(4-methoxyphenylsulfonamido)naphthalen-1-yl)phenylsulfonamido)acetate (20)

To a solution of compound **19** (1.2 g, 2.70 mmol) in EtOH (20 mL) was added SnCl_2_·2H_2_O (3.0 g, 13.50 mmol) and the reaction was stirred at 70 °C for 45 min. The mixture was diluted with H_2_O, basified to pH 7–8 with sat. NaHCO_3_ and filtered through Celite. The filtrate was extracted with EtOAc (4 x 50 mL) and the combined organic layers were washed with sat. NaHCO_3_ (3 x 100 mL), H_2_O (3 x 100 mL), sat. brine (3 x 100 mL) and dried over anhydrous Na_2_SO_4_. The crude product was used in the next step without further purification.

To a solution of the above crude product in DCM (50 mL) was added 4-methoxybenzenesulfonyl chloride (614 mg, 2.97 mmol) and pyridine (330 μL, 4.05 mmol) and the reaction was stirred at rt for 16 h. On completion, hexane (30 mL) was added and the resulting precipitate was collected by filtration and washed with DCM/hexane (1:1 v/v). The crude product was purified by flash chromatography (EtOAc/Hexane 4:6 v/v) to afford **20** (1.1 g, 70 %) as an off-white powder. ^1^H NMR (400 MHz, CDCl_3_) *δ* (ppm): 8.20–8.13 (m, 1H), 7.82–7.75 (m, 1H), 7.73–7.65 (m, 2H), 7.65–7.55 (m, 2H), 7.55–7.43 (m, 2H), 7.26 (d, *J* = 8.0 Hz, 3H), 7.10 (d, *J* = 8.0 Hz, 1H), 6.94–6.82 (m, 5H), 6.98–6.75 (m, 5H), 6.95–6.78 (m, 5H), 4.64–4.52 (m, 1H), 4.27 (dd, *J* = 17.7, 8.7 Hz, 1H), 4.15–4.02 (m, 2H), 3.84 (s, 3H), 3.81 (s, 3H), 1.15 (t, *J* = 7.1 Hz, 3H). ^13^C NMR (100 MHz, CDCl_3_) *δ* (ppm): 168.3, 163.4, 134.5, 133.0, 132.7, 130.8, 130.4, 130.3, 129.6, 129.3, 127.7, 127.4, 127.3, 124.94, 121.6, 120.5, 114.3, 114.0, 61.5, 55.7, 55.5, 53.4, 14.4. The above data are in agreement with those previously reported in the literature [18].

##### 2.3.15 *tert*-Butyl 2-(*N*-(4-(*N*-(2-ethoxy-2-oxoethyl)-4-methoxyphenylsulfonamido)naphthalen-1-yl)-4-methoxyphenylsulfonamido)acetate (21)

Prepared analogously to compound **19** from **18** (150 mg, 0.26 mmol) and *tert*-butyl bromoacetate (50 μL, 0.32 mmol). The crude product was purified by flash chromatography (EtOAc/hexane 3:7 v/v) to afford **21** (120 mg, 65 %) as a white powder. ^1^H NMR (500 MHz, DMSO-*d*_6_) *δ* (ppm): 8.36–8.26 (m, 1H), 8.24–8.11 (m, 1H), 7.69–7.48 (m, 6H), 7.20–6.99 (m, 5H), 6.90–6.82 (m, 1H), 4.60–4.21 (m, 4H), 4.12–3.98 (m, 2H), 3.86 (s, 3H), 3.84 (s, 3H) 1.24 (s, 9H), 1.15–0.96 (m, 3H). ^13^C NMR (125 MHz, DMSO-*d*_6_) *δ* (ppm): 168.5, 168.3, 167.3, 167.2, 162.9, 162.8, 137.7, 137.5, 136.9, 136.8, 133.1, 132.5, 132.4, 132.2, 130.4, 129.5, 129.4, 129.3, 128.8, 128.7, 126.9, 126.7, 126.6, 126.5, 126.4, 126.1, 125.4, 124.7, 124.6, 124.5, 124.4, 114.4, 114.3, 114.2, 81.5, 81.4, 60.8, 55.8, 55.7, 54.1, 53.6, 53.3, 53.2, 27.4, 13.9, 13.7. LC-MS: *m/z* (ESI) = 737.00 [M+K]^+^, t_R_ = 7.68 min, >95 %.

##### 2.3.16 2-(*N*-(4-(*N*-(2-Ethoxy-2-oxoethyl)-4-methoxyphenylsulfonamido)naphthalen-1-yl)-4-methoxyphenylsulfonamido)acetic acid (22)

A solution of **21** (642 mg, 0.92 mmol) in DCM (5 mL) was cooled to 0 °C and TFA (5 mL) was added dropwise over a period of 2 min and stirring was continued for 1 h. The solvent was removed under reduced pressure and the crude product was purified by flash chromatography (EtOAc/hexane/formic acid 50:49:1 v/v/v) to afford **22** (350 mg, 59 %) as a white solid. ^1^H NMR (500 MHz, DMSO-*d*_6_) *δ* (ppm): 13.03–12.67 (br, 1H), 8.34–8.26 (m, 1H), 8.20–8.13 (m, 1H), 7.64–7.53 (m, 6H), 7.11–6.84 (m, 6H), 4.55–4.39 (m, 4H), 4.09–3.94 (m, 2H), 3.94–3.77 (m, 6H), 1.07 (m, 3H). ^13^C NMR (125 MHz, DMSO-*d*_6_) *δ* (ppm): 169.9, 169.8, 168.5, 162.8, 162.5, 162.4, 162.3, 137.9, 137.6, 136.9, 136.3, 133.0, 132.9, 132.8, 132.7, 130.4, 130.2, 129.9, 129.8, 129.5, 129.4, 128.9, 128.6, 128.5, 128.2, 126.8, 126.6, 126.3, 125.2, 124.9, 124.5, 124.9, 124.5, 114.6, 114.3, 114.1, 60.9, 60.8, 55.7, 55.2, 53.4, 53.3, 53.1, 53.0. HRMS (ESI): calculated for C_30_H_30_N_2_O_10_S_2_ [M+H]^+^ 643.1420, found 643.1408. HPLC retention time: t_R_ = 10.09 min, purity: >95 %.

##### 2.3.17 2-(*N*-(4-(*N*-(2-Amino-2-oxoethyl)-4-methoxyphenylsulfonamido)naphthalen-1-yl)-4-methoxyphenylsulfonamido)acetic acid (23)

To a solution of **22** (110 mg, 0.17 mmol) in DMF (1 mL) were added successively pyridine (14 μL, 0.178 mmol), Boc_2_O (56 mg, 0.26 mmol) and (NH_4_)_2_CO_3_ (13 mg, 0.13 mmol) and the reaction mixture was stirred for 24 h at rt. On completion, H_2_O (10 mL) was added and the resulting precipitate was collected by filtration, washed with H_2_O and ice-cold Et_2_O and dried *in vacuo*. The crude ester was hydrolysed according to the general method C. After acidification and filtration, the product was washed successively with H_2_O, Et_2_O and DCM and dried overnight in a vacuum desiccator to afford **23** (34 mg, 76 %) as a white powder. ^1^H NMR (500 MHz, DMSO-*d*_6_) *δ* (ppm): 13.15–12.15 (br, 1H), 8.24 (m, 2H), 7.63–7.49 (m, 6H), 7.33–7.25 (br, 2H), 7.20–6.75 (m, 10H), 4.58–4.05 (m, 4H), 3.86–3.79 (m, 6H). ^13^C NMR (125 MHz, DMSO-*d*_6_) *δ* (ppm): 169.8, 168.7, 162.8, 137.0, 136.9, 133.7, 132.9, 130.3, 130.2, 130.1, 129.8, 129.7, 129.2, 129.1, 126.5, 126.3, 126.2, 124.1, 124.0, 114.7, 114.2, 114.0, 55.5, 54.7, 53.1, 53.0. LC-MS: *m/z* (ESI) = 1224.15 [2 M−H]^−^, t_R_ = 5.56 min, >95 %. HRMS (ESI): calculated for C_28_H_27_N_3_O_9_S_2_ [M−H]^−^ 612.1110, found 612.1093. HPLC retention time: t_R_ = 9.33 min, purity: >95 %.

##### 2.3.18 Ethyl 2-(*N*-(4-(*N*-(cyanomethyl)-4-methoxyphenylsulfonamido)naphthalene-1-yl)-4-methoxyphenylsulfonamido)acetate (24)

Prepared analogously to compound **19** from **18** (143 mg, 0.24 mmol) and bromoacetonitrile (10 μL, 0.14 mmol). The crude product was purified by flash chromatography (EtOAc/hexane 3:7 v/v) to afford **113** (85 mg, 57 %) as a white powder. ^1^H NMR (400 MHz, DMSO-*d*_6_) *δ* (ppm): 8.39–8.17 (m, 1H), 8.13–7.94 (m, 1H), 7.75–7.54 (m, 6H), 7.24–6.84 (m, 6H), 5.10–4.84 (m, 2H), 4.62–4.41 (m, 2H), 4.10–3.94 (m, 2H), 3.94–3.80 (m, 6H), 1.08 (t, *J* = 7.3 Hz, 3H). LC-MS: *m/z* (ESI) = 625.20 [M+H]^+^, t_R_ = 6.99 min, purity: >95 %.

##### 2.3.19 *N*-(4-((*N*-((1*H*-tetrazol-5-yl)methyl)-4-methoxyphenyl)sulfonamido)naphthalen-1-yl)-*N*-((4-methoxyphenyl)sulfonyl)glycine (25)

To a solution of **24** (83 mg, 0.13 mmol) in DMF (2 mL), was added NaN_3_ (9 mg, 0.34 mmol) and NH_4_Cl (23 mg, 0.43 mmol) and the reaction was stirred at 100 °C for 24 h. On completion, the reaction mixture was diluted with H_2_O (20 mL), extracted with EtOAc (3 x 20 mL) and the combined organic layers were washed with H_2_O (3 x 80 mL), sat. brine (3 x 80 mL), dried over anhydrous MgSO_4_ and evaporated to dryness under reduced pressure. The crude ester was hydrolysed according to the general method C. Purification by flash chromatography (DCM/EtOAc/formic acid 80:19:1 v/v/v) afforded **25** (31 mg, 37 %) as a white solid. ^1^H NMR (500 MHz, DMSO-*d*_6_) *δ* (ppm): 13.01–12.30 (br, 2H), 8.33–7.99 (m, 2H), 7.67–7.49 (m, 5H), 7.40 (d, *J* = 8.8 Hz, 1H), 7.20–7.09 (m, 3H), 7.04–6.74 (m, 3H), 5.40–4.92 (m, 2H), 4.55–4.37 (m, 2H), 3.96–3.83 (m, 6H). ^13^C NMR (125 MHz, DMSO-*d*_6_) *δ* (ppm): 170.1, 169.0, 163.6, 163.1, 162.4, 162.3, 137.3, 137.2, 136.3, 135.7, 133.7, 132.8, 132.2, 132.1, 130.8, 130.7, 130.5, 129.9, 128.8, 128.7, 128.4, 128.2, 126.9, 126.7, 126.5, 126.2, 125.9, 124.8, 124.7, 123.8, 123.0, 114.5, 114.3, 114.1, 114.0, 55.4, 55.2, 55.1, 55.0, 53.2, 53.1. LC-MS: *m/z* (ESI) = 1275.25 [2 M−H]^−^, t_R_ = 5.78 min, >95 %. HRMS (ESI): calculated for C_28_H_25_N_6_O_8_S_2_ [M − H]^−^ 637.1176, found 637.1147. HPLC retention time: t_R_ = 8.47 min, purity: >95 %.

#### 2.4 Biological, biophysical and computational methods

##### 2.4.1 Keap1 protein expression and purification

Expression of the Keap1 Kelch domain was performed as described previously [23,31,32].

##### 2.4.2 Fluorescence polarisation (FP) assays

The FP assays were carried out according to previously described methods [23,[31], [32], [33], [34]]. Briefly, varying concentrations of the small molecule inhibitor dissolved in DMSO were plated into untreated Corning® black 96 well plates containing a solution of the Keap1 Kelch domain (200 nM final concentration) and the fluorescent peptide FITC-β-DEETGEF-OH (1 nM final concentration) in Dulbecco's phosphate buffered saline (DPBS) at pH 7.4 (11 % final DMSO concentration, 100 μL final volume). Following incubation under slow agitation (30 min at rt in the absence of light), the plates were transferred to a PHERAstar microplate reader and the FP was recorded. All measurements were recorded in triplicate. The data were normalised to the control and then fitted to a standard four-parameter logistic function using the Origin Pro software.

##### 2.4.3 Differential scanning fluorimetry (DSF) assays

The DSF assays were carried out according to the previously described method [23]. Solutions of the Keap1 Kelch domain protein (5 μM final concentration) and the small molecule inhibitor (10 μM final concentration) were prepared in DPBS at pH 7.4 (10 % final DMSO concentration, 20 μL final volume) in an Eppendorf tube. The solution was centrifuged briefly (1500 rpm, 60 s) to remove any particulates and mix the samples, followed by a 30 min incubation at RT. The samples were loaded into Tycho NT.6 capillaries (Nanotemper) in triplicate and positioned in the Tycho NT 1.6 instrument. The samples were heated from 35 °C to 95 °C using the default settings and the ratio of the intrinsic tyrosine and tryptophan fluorescence intensities (330 and 350 nm) for each capillary tube was recorded. The fluorescence ratio vs temperature was plotted and the inflection temperature (T_i_) of the profile was recorded. Changes in inflection (ΔT_i_) for the inhibitors were calculated by subtracting the mean T_i_ of the Keap1 sample plus vehicle from the mean Keap1 plus binder value (n = 3).

##### 2.4.4 Isothermal titration calorimetry (ITC) assays

The measurements were performed as described previously [23,31]. The small molecule inhibitor at 500 μM was titrated over 30 injections against 50 μM protein in buffer containing 25 mM HEPES (pH 7.4), 200 mM NaCl and 5 % DMSO. ITC experiments were performed using a MicroCal PEAQ-ITC instrument (Malvern Instruments, UK) at 25 °C with a stirring speed of 750 rpm. The first injection volume was of 0.3 μL followed by 29 injections of 1.3 μL and a gap of 120 s between each injection. Data analyses were performed as previously described. Each small molecule ITC measurement was performed in duplicate and resultant values obtained were averaged after independent analysis.

##### 2.4.5 Microscale thermophoresis (MST) assays

Keap1 was labelled using the Protein Labelling Kit RED-NHS 2nd Generation (NanoTemper, München, Germany). Keap1 was diluted to 2 μM in RED-NHS Labelling Buffer. 10 μg of RED-NHS 2nd Generation labelling dye was freshly dissolved in 25 μL of DMSO to yield a concentration of 600 μM, then further diluted in RED-NHS Labelling Buffer to 300 μM. A volume of 20 μL of the 300 μM labelling dye was thoroughly mixed with 180 μL of 2 μM Keap1 and incubated at rt in a dark environment for 3 h. The labelled sample was subsequently purified using the B-column pre-equilibrated with Keap1 MST buffer consisting of 50 mM HEPES, pH 7.5, 200 mM NaCl and 0.05 % Pluronic F-127. The sample was transferred to the centre of the resin bed in the B-column. 550 μL of Keap1 MST buffer was pipetted into the B-column and allowed to enter completely, followed by elution of the sample with 450 μL of Keap1 MST buffer and the flow-through was collected into a 1.5 mL Eppendorf tube. The collected sample was centrifuged at 15,000×*g* for 10 min at 4 °C to remove aggregates. Protein concentration in the collected sample was determined using the NanoDrop spectrophotometer. The sample was then tested in MST pre-test to evaluate the labelling quality.

Four sets of MST measurements were conducted in triplicate to quantify the binding affinities between Keap1 and compounds **2**, **13** and **25**. Labelled Keap1 was diluted to 20 nM in Keap1 MST buffer prior to the reaction setup. Compounds **2**, **13** and **25** were initially diluted to 16 μM, 12 μM and 6 μM, respectively, in Keap1 MST buffer, and then serially diluted 15 times in the same buffer. An equal volume of 20 nM Keap1 was thoroughly mixed with each diluted sample and incubated for 30 min at rt. For the interaction between Keap1 and **25**, which exhibited a lower *K*_d_ value, labelled Keap1 was diluted to 2 nM in Keap1 MST buffer prior to the reaction setup. Compound **25** was initially diluted to 100 nM in Keap1 MST buffer and then serially diluted 15 times in the same buffer. An equal volume of 2 nM Keap1 was mixed with each diluted sample and incubated at rt for 30 min. Incubated samples were loaded into Monolith NT.115 standard capillaries and measured using the Monolith NT.115^Pico^ instrument in triplicate. Parameters and measurements were set and controlled by the MO.Control software and collected datasets were analysed using the MO.Affinity Analysis software.

##### 2.4.6 X-ray crystallography and structure determination of Keap1 binder complexes

The measurements were performed as described previously [23]. Crystals for the Keap1-Kelch domain in complex with **13** or **25** appeared after 2–3 days in 3.7 M sodium formate, pH 7.0 at 4 °C. Compound **25** was also crystallised with Keap1 at 18 °C. The crystals were further optimized by streak seeding to obtain single crystals for diffraction measurements. The single crystals of the complex were cryo-protected with 20 % w/v ethylene glycol in 1.2-fold of the crystallisation solution and flash-frozen in liquid nitrogen.

Diffraction data for individual crystals were collected at beamline I03 at Diamond Light Source. Data were processed using either XDS46 or iMosflm [35] and scaled to resolutions as mentioned in Table S1. The structures of the complexes were solved by molecular replacement using the native Keap1 structure (PDB entry 1ZGK) [36] as a search model. The structures were manually refined using Coot [37] followed by Phenix [38]. The crystallographic statistics are given in Table S1. Crystals of the binary Keap1-Kelch domain in complex with the compounds contain one molecule in the asymmetric unit.

##### 2.4.7 NMR methods

NMR data were acquired on a Bruker NEO spectrometer equipped with a QCI-F cryoprobe operating at a ^1^H Larmor frequency of 600 MHz, and a Bruker Avance III spectrometer equipped with a QCI-P cryoprobe operating at a ^1^H Larmor frequency of 700 MHz. Data were acquired and processed using Topspin 4.2.0 and 3.5pl6 respectively and analysed in Julia using NMRTools.jl (v0.1.12) [39].

A series of 1D ^1^H NMR spectra were acquired for samples of compounds **2**, **13** and **25** dissolved in d_6_-DMSO at 300, 310 and 320 K (600 MHz, zg30, 1 s recycle delay, 1.4 s acquisition time, 20 ppm spectral width). A sample of compound **25** was also prepared in PBS in D_2_O (pH∗ ∼7.4), and similar 1D ^1^H experiments were acquired (600 MHz, zgpr, 3 s recycle delay, 1.4 s acquisition time, 20 ppm spectral width), and referenced according to the water chemical shift. Spectra were processed without apodisation and the methoxy singlet region (approximately 3.75–3.95 ppm) was selected for lineshape fitting. Exchange was quantified using a two-site exchange model [40] implemented in Julia, where resonances were fitted to calculate chemical shifts of the two states and the exchange rates *k*_AB_ and *k*_BA_ between them. Lorentzian lineshapes were assumed with an exchange-free transverse relaxation rate of 3 s^−1^ (DMSO) and 4 s^−1^ (D_2_O). Reported uncertainties were determined from the standard errors of the fits.

A series of pseudo-3D EXSY spectra were then acquired for compound **25** in D_2_O at temperatures of 280, 290, 300, 310 and 320 K (700 MHz, pulse sequence adapted from noesygpphpr and available on request, 1 s recycle delay, 13 mixing times from 5 to 1000 ms, acquisition times of 367 ms and 52 ms and spectra widths of 16 ppm and 3.5 ppm for direct and indirect dimensions respectively). Spectra were processed with linear prediction and cosine-squared window functions. For each temperature, methoxy singlet resonances were selected for analysis, and the intensities of the diagonal ($I_{AA}$ and $I_{BB}$) and cross peaks ($I_{AB}$ and $I_{BA}$) were fitted to Eq. (1) as a function of the mixing time, *T*, to determine the exchange rates $k_{AB}$ and $k_{BA}$ [41]. The ‘A’ state was defined to be the more shielded signal. Reported uncertainties were obtained from the non-linear fitting procedure.

(1) $$\begin{array}{c} \left(\begin{array}{cc} I_{AA} & I_{AB}\\ I_{BA} & I_{BB} \end{array}\right)\left(T\right)=\exp\left[\left(\begin{array}{cc} -R_{1,A}-k_{AB} & k_{BA}\\ k_{AB} & -R_{1,B}-k_{BA} \end{array}\right)T\right]\cdot \left(\begin{array}{cc} I_{A}^{0} & 0\\ 0 & I_{B}^{0} \end{array}\right) \end{array}$$

Exchange rates $k_{AB}$ and $k_{BA}$ were used to determine the relative populations $p_{A}=\frac{k_{BA}}{k_{AB}+k_{BA}}$ and $p_{B}=\frac{k_{AB}}{k_{AB}+k_{BA}}$ and were fitted to van't Hoff and Eyring equations (with unit transmission coefficient) to estimate the enthalpy and entropy changes and enthalpy and entropy of activation.

##### 2.4.8 Molecular dynamics (MD) simulations

Molecular dynamics simulations were run using the AMBER18 software suite [42]. PDB files of the Keap1 Kelch domain in complex with **25** were stripped of solvent and ions then protonated according to their AMBER atom types using xleap. The complexes were parameterised for MD using the FF14SB (protein, xleap) and GAFF (small molecule, antechamber) atom types and forcefields. The complexes were neutralised with K^+^ ions then solvated in TIP3P water box that extended 10 Å from the protein. Potassium chloride was added to final concentration of 150 mM. The resulting complexes were energy minimised then heated to the simulation temperature (277 K or 300 K) using an 8-step procedure with progressive relaxation of the positional restraints from 100 to 0 kcal mol^−1^ Å^−2^ as previously described [43]. Unrestrained MD was run for 10 ns to equilibrate the system then production MD was run for 1 μs. Each simulation was repeated three times using different initial velocity distributions. Simulations were also performed of cis and *trans*-forms of **25** alone at 300 K over 500 ns. Interaction energies were calculated using MMGBSA and esander calculations for each frame of the trajectory. Representative time-averaged structures were produced from the trajectories and RMSD calculations were performed using cpptraj. Trajectories were visualised using VMD 1.9.3 [44] and images were produced using UCSF ChimeraX 1.7 [9].

##### 2.4.9 NQO1 induction assays

The method using Hepa1c1c7 cells has been described previously [23]. Briefly, cells were seeded into 96-well plates (2 × 10^4^ cells/200 μL per well) then incubated overnight at 37 °C. The cells were treated with compound (10 μM final concentration) or vehicle (final DMSO concentration 0.1 % DMSO) and incubated for a further 24 h or 48 h. The culture medium was aspirated and the cells treated with lysis buffer (50 μL per well, 0.1 % Tween 20 in 2 mM EDTA (pH 7.5)) then agitated for 15 min. Enzyme reaction mixture was added to each well (200 μL of 25 mM Tris buffer (pH 7.5) containing BSA (0.067 %), Tween20 (0.01 %), FAD (5 μM), G6P (1 mM), NADP (30 μM), G6P dehydrogenase (40 units), MTT (0.03 %), menadione (50 μM)) for 5 min then stop solution was applied (40 μL of 10 % SDS, final concentration 1.5 % SDS). Following brief agitation of the plate, the absorbance at 595 nm was measured. The background optical density was measured from wells containing tissue culture medium, lysis buffer, enzyme and stop solutions in the absence of Hepa1c1c7 cells. The change in background-corrected NQO1 activity (change in optical density) relative to the control was determined for each compound.

##### 2.4.10 Nrf2 luciferase reporter assay

The induction of luciferase activity in the stably transfected MCF7 ARE-luciferase reporter cell line was measured using an adaptation of a published procedure [45]. Cells were seeded in tissue-culture treated 96 well plates with a density of 3 x 104 cells per 200 μL per well and incubated at 37 °C overnight. The cells were treated with compound or vehicle control (1 % final DMSO concentration) and incubated for a further 24 h. The culture medium was aspirated and the cells were washed with 100 μL per well PBS. The cells were lysed using 75 μL per well lysis buffer (0.2 % Triton X-100 in 25 mM Tris pH 7.5) under gentle agitation at rt for 30 min. The samples were transferred to 500 μL Eppendorf tubes and centrifuged at 10000 g for 5 min at 4 °C. 50 μL of supernatant from each sample were transferred to a new 96 well plate. 50 uL of working reagent (100 mM Tris pH 8.0, 5 mM MgCl_2_, 100 μM coenzyme A, 80 μM luciferin, and 5 mM ATP) were added to each well and the luciferase activity was measured.

#### 2.4.11 Keap1-Glow Cellular Thermal Shift Assay (CETSA)

U2OS FRT/TO cells with doxycycline-inducible expression of Keap1-mCherry or mCherry were treated with 0.5 μg/mL of doxycycline for 72–96 h to induce the expression of the fluorescent proteins. Lysates of U2OS cells with doxycycline-inducible expression of Keap1-mCherry or mCherry were prepared by lysing in DPBS with EDTA-free protease inhibitor cocktail (PIC) using at least 22 strokes of the Dounce homogenizer. The lysates were cleared by centrifugation at 9600×*g* for 5 min at 22 °C and incubated with 10 μM compound **25** or vehicle (0.4 % DMSO) for 1 h at 37 °C [46]. The lysates were then heated for 3 min at the indicated range of temperatures (39 °C–72 °C, with 3 °C increments) using a thermal cycler, and the aggregated material was removed by a high-speed centrifugation at 17,000×*g* at 4 °C for 40 min or 15,000×*g* at 4 °C for 1 h. The soluble fractions were transferred to 96-well white opaque plates, and the fluorescence (580 nm excitation wavelength (20 nm excitation bandwidth), 635 nm emission wavelength (35 nm emission bandwidth)) was measured using a plate reader (TECAN Spark 10 M).

#### 2.4.12 Immunoblotting

Hepa1c1c7 cells (5 × 10^5^ cells/well, n = 3) were seeded on six-well plates, and on the next day were treated with compound **25** (10 μM or 50 μM) or vehicle (0.1 % DMSO) for 6 h. Cells were washed twice in PBS, and lysed in 200 μL of SDS lysis buffer containing 2 % SDS, 62.5 mM Tris-HCl pH 6.8 and 10 % glycerol. The cell lysates were boiled for 3 min, sonicated for 25 s (25 %, 4 s on, 1 s off), supplemented with DTT up to 0.1 M and Bromophenol Blue, and snap-frozen. The lysates were thawed, and 20 μg of total protein was separated on a pre-cast NuPage gel under MOPS buffer, transferred onto nitrocellulose membrane and blotted with *anti*-Nrf2 (Cell Signaling Technology, clone D1Z9C, 1:1000 dilution), anti-HO1 (Abcam, ab13243, 1:2000 dilution) or anti-alpha tubulin (Abcam, clone EP1332Y, 1:5000 dilution) in 5 % milk in PBS with 0.1 % Tween, followed by appropriate secondary antibodies (LiCor BioSciences, 1:15000 dilutuon) and scanned using Odyssey CLx Imaging System.

#### 2.4.13 Gene expression analysis

Hepa1c1c7 cells (5 × 10^5^ cells/well, n = 3) were seeded on six-well plates, and on the next day were treated with compound **25** (10 μM or 50 μM) or vehicle (0.1 % DMSO) for 16 h. After two washes with PBS, the cells were lysed. RNA was then extracted, cDNA was synthesised, and the levels of mRNA for each gene were determined by real-time quantitative PCR (TaqMan). The levels of 18S mRNA in each sample were used as a control for normalization.

## 3 Results and discussion

### 3.1 Design and Synthesis

A series of naphthalene *bis*-sulfonamides were synthesised with N-alkyl substituents of varying polarity, size and pKa (Table 1). The compounds were selected for synthesis in symmetrical form in which the two N-alkyl substituents were equivalent. Subsequently a subset of the compounds were synthesised in an asymmetric form in which one N-alkyl substituent was a methylene carboxylate and the second substituent contained either an ester, amide or tetrazole.

**Table 1:**
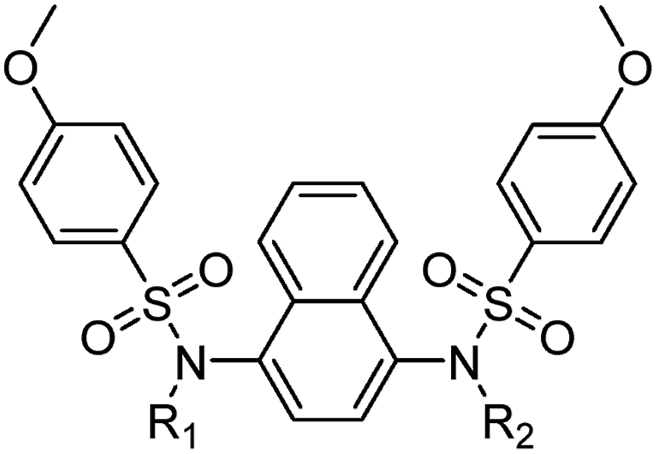
Keap1 binding and NQO1 induction by symmetric and asymmetric naphthalene sulfonamides.

Compound	R_1_	R_2_	FP IC_50_ (nM)	DSF ΔT_i_ (°C)^a^	NQO1-fold induction (@ 10 μM)
Symmetric					
**11**	CH_2_CO_2_Et	CH_2_CO_2_Et	0 %^b^	0.5 ± 0.5	ND^c^
**2**	CH_2_CO_2_H	CH_2_CO_2_H	59 ± 6^d^	21.9 ± 0.2	2.34 ± 0.35
**4**	CH_2_CONH_2_	CH_2_CONH_2_	231 ± 6	7.6 ± 0.1	0.94 ± 0.17
**12**	CN	CN	0 %	1.0 ± 0.2	0.89 ± 0.24
**13**	CH_2_Tet^e^	CH_2_Tet	76 ± 3	20.4 ± 0.1	1.94 ± 0.22
**17**	CH_2_Ox^f^	CH_2_Ox	0 %	0.6 ± 0.1	1.25 ± 0.18
**Asymmetric**					
**22**	CH_2_CO_2_Et	CH_2_CO_2_H	0 %	ND	0.77 ± 0.18
**23**	CH_2_CONH_2_	CH_2_CO_2_H	143 ± 18	10.0 ± 0.2	1.90 ± 0.05
**25**	CH_2_Tet	CH_2_CO_2_H	31 ± 2	19.4 ± 0.4	2.01 ± 0.05

a Keap1 vehicle control T_i_ = 61.3 ± 0.3 °C.

b Percentage inhibition at 10 μM concentration of inhibitor.

c Not determined.

d Reference 20.

e Tet = tetrazol-5-yl.

f Ox = 5-methyl-1,3,4-oxadiazol-2-yl.

**Scheme 1:**
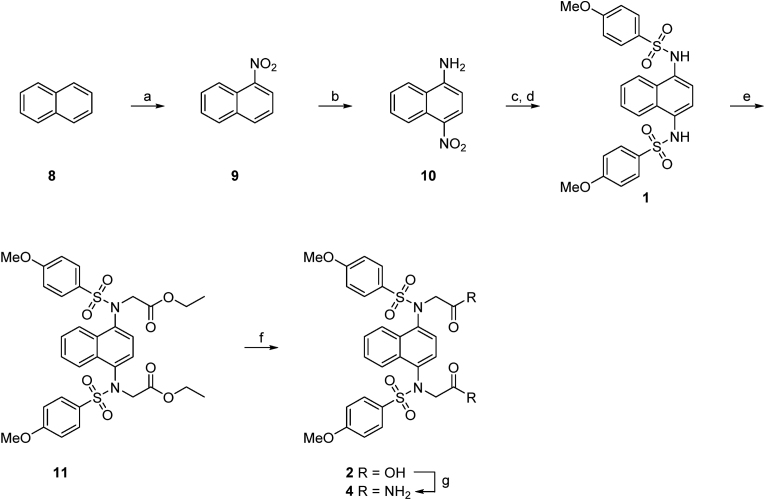
**Synthesis of *bis*-acid naphthalene 2.** Reagents and conditions: a) KNO_3_, TFAA, CHCl_3_, rt, 5 h, 91 %; b) NH_2_OH·HCl, KOH, EtOH/MeOH, 60 °C, 2 h, 73 %; c) SnCl_2_·2H_2_O, EtOH, 60 °C, 2 h; d) 4-methoxybenzenesulfonyl chloride, pyridine, DMAP, DCM, rt, 16 h, 49 %; e) ethyl bromoacetate, K_2_CO_3_, DMF, rt, 4 h, 74 %; f) 1n NaOH, THF/MeOH (1:1 v/v), rt, 2 h, 65 %; g) Boc_2_O, pyridine, (NH_4_)_2_CO_3_, DMF, Ar, 0 °C to rt, 19 h, 77 %.

**Scheme 2:**
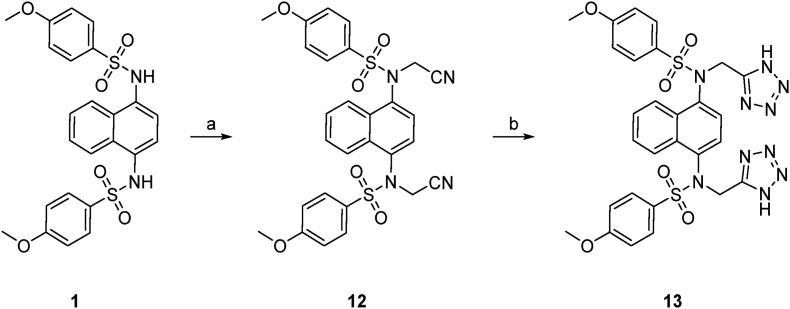
**Synthesis of *bis*-nitrile 12 and *bis*-1*H*-tetrazole 13.** Reagents and conditions: a) bromoacetonitrile, K_2_CO_3_, DMF, rt, 4 h, 67 %; b) NaN_3_, NH_4_Cl, DMF, 100 °C, 16 h, 31 %.

**Scheme 3:**
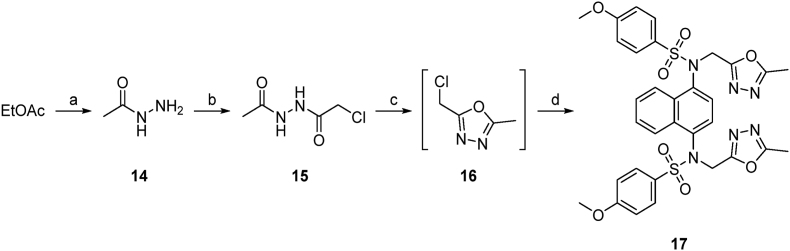
**Synthesis of oxadiazole-containing compound 17.** Reagents and conditions: a) Hydrazine hydrate, EtOH, reflux, 24 h, 81 %; b) chloroacetyl chloride, Na_2_CO_3_, H_2_O, 0 °C to rt, 90 min, 45 %; c) POCl_3_, anhydrous MeCN, Ar, 0 °C–80 °C, 3 h; d) **1**, K_2_CO_3_, DMF, rt, 8 h, 47 %.

The symmetrical compounds were synthesised from naphthalene **8** via nitration to give **9** followed by amination to give 4-nitronaphthylamine **10** (Scheme 1). This was reduced to an intermediated *bis*-amine, then treated with two equivalents of 4-methoxybenzenesulfonyl chloride to give the symmetrical *bis*-sulfonamide **1**. *N*-Alkylation of the sulfonamide with ethyl bromoacetate followed by ester hydrolysis of **11** gave the *bis*-acid **2**. The acid **2** was converted to the amide **4** by activation with Boc anhydride, followed by treatment with ammonium carbonate. The *bis*-tetrazole **13** was formed by treatment of the *bis*-sulfonamide **1** with bromoacetonitrile to give the *bis*-cyano compound **12**, that was treated with sodium azide to form the corresponding tetrazole **13** (Scheme 2). The oxadiazole-substituted compound was synthesised by reacting acetohydrazine **14** with chloroacetyl chloride to give the *N′*-acetyl-2-chloroacetohydrazide **15**. After cyclization the crude chloromethyloxadiazole **16** was used to synthesise **17** (Scheme 3).

**Scheme 4:**
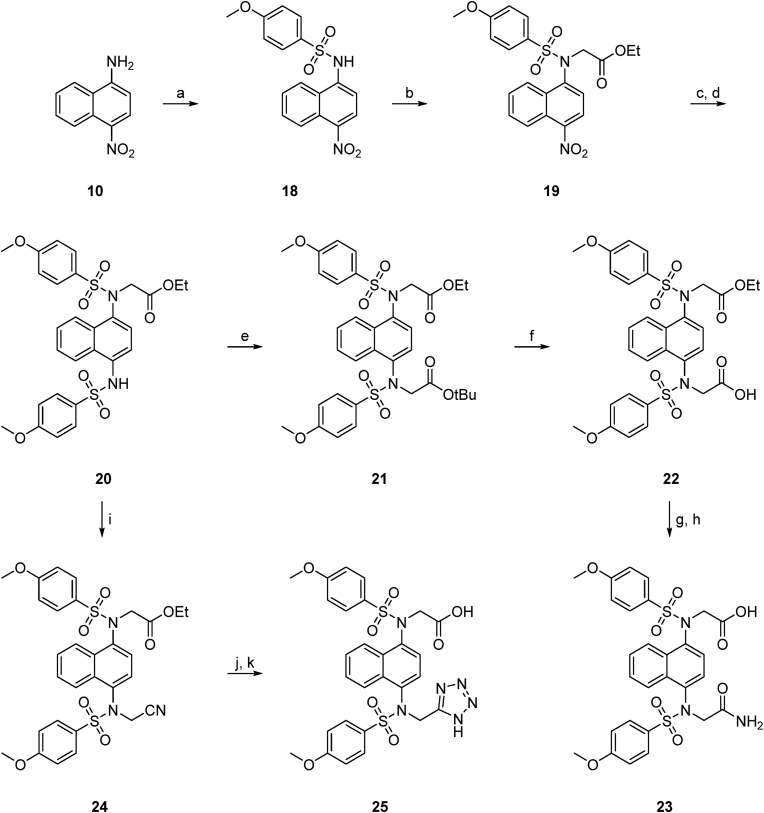
**Synthetic route to de-symmetrised analogues 22–25.** Reagents and conditions: a) 4-methoxybenzenesulfonyl chloride, toluene/pyridine (1:1 v/v), 100 °C, 2 h, 68 %; b) ethyl bromoacetate, K_2_CO_3_, DMF, rt, 24 h, 45 %; c) SnCl_2_·2H_2_O, EtOH, 70 °C, 45 min; d) 4-methoxybenzenesulfonyl chloride, pyridine, DMAP, DCM, rt, 16 h, 70 %; e) *tert*-butyl bromoacetate, K_2_CO_3_, DMF, rt, 4 h, 65 %; f) TFA/DCM (1:1 v/v), 0 °C to rt, 1 h, 59 %; g) Boc_2_O, pyridine, (NH_4_)_2_CO_3_, DMF, Ar, 0 °C to rt, 24 h; h) 1 n NaOH, THF/MeOH (1:1 v/v), 2 h, 50 %; i) bromoacetonitrile, K_2_CO_3_, DMF, rt, 6 h, 57 %; j) NaN_3_, NH_4_Cl, DMF, 100 °C, 24 h; k) 1n NaOH, THF/MeOH (1:1 v/v), 2 h, 37 %.

The asymmetric analogues were synthesised by first reaction of **10** with 4-methoxybenzenesulfonyl chloride and ethyl bromoacetate to give the sulfonamide **19** (Scheme 4). Reduction of the nitro group followed by sulfonamide formation and N-alkylation with *t*-butyl bromoacetate to give **21** or with bromoacetonitrile to give **24** allowed the synthesis of the corresponding acid ester **22**, acid/amide **23**, and acid/tetrazole **25**.

### 3.2 Structure-activity relationships

The binding of the symmetrical analogues **2**, **4**, **11**–**13** and **16** to Keap1 measured using a fluorescence polarisation competition assay was consistent with previous observations [13,15,23]. The *bis*-carboxylic acid **2** was superior to the amide **4**, ester **11** and cyano **12** compounds (Table 1). The tetrazole **13** had similar activity to the *bis*-acid **2** (IC_50_ 76 nM cf. 59 nM respectively), consistent with its role as a carboxylate bioisostere, however the corresponding 1,3,4-oxadiazole **16** was not tolerated. The asymmetric analogues incorporated a single carboxylic acid and had binding activity that was intermediate between the parent symmetrical structures in the case of the amide **23**. The ester containing molecule **22** was inactive. However, the acid/tetrazole compound **25** had an IC_50_ that was ∼2-fold lower than the *bis*-acid **2** (IC_50_ 31 nM cf. 59 nM respectively) and ∼2.5-fold lower than the *bis*-tetrazole **13** (IC_50_ 76 nM).

The interaction between the Keap1 Kelch domain and the test compounds was also evaluated using a differential scanning fluorimetry (DSF) thermal shift assay [23]. The magnitude of the change in inflection temperature (ΔT_i_) at a concentration of 10 μM was consistent with the trends in measured FP IC_50_ values. Compounds with IC_50_s in the range <100 nM had ΔT_i_s > 19 °C (**2**, **13**, **25**), those with IC_50_s between 100 and 1000 nM had ΔT_i_s between 5 and 10 °C (**4**, **10**) and those with higher IC_50_ values had ΔT_i_s between 0 and 2 °C (**11**, **12**, **16**).

**Fig. 2:**
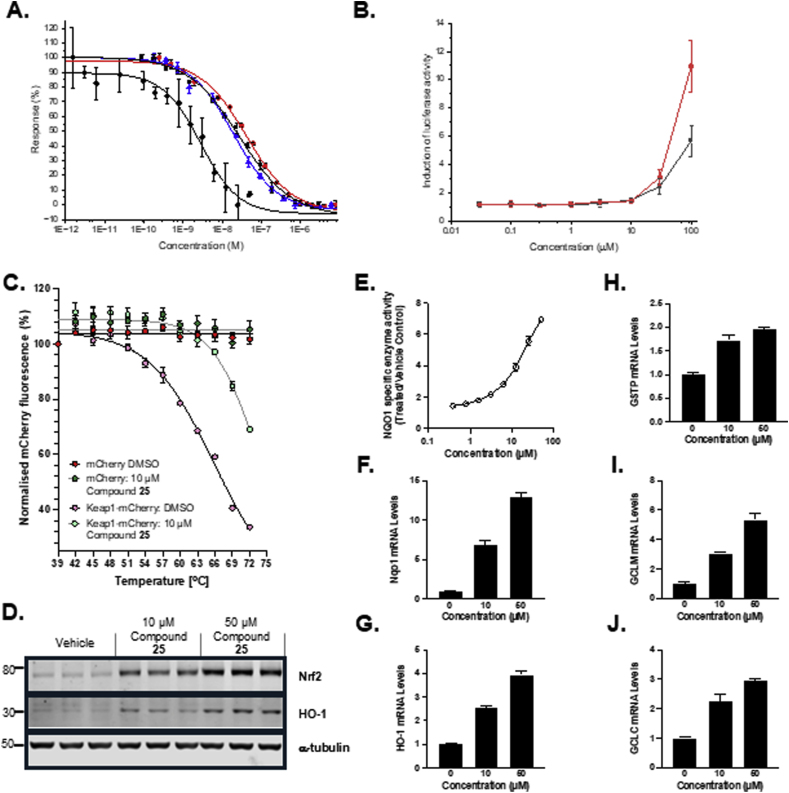
A. Binding profiles of compounds **2** (red ●), **13** (blue ▲) and **25** (black ■) to Keap1 determined by MST. Compound **25** was also tested using a 1 nM Keap1 concentration (black ♦). B. Induction of luciferase activity in MCF7 cells relative to vehicle, compounds **2** (red ●) and **25** (black ■). C. mCherry fluorescence intensity in soluble fractions of lysates of Keap1-mCherry, or mCherry-expressing U2OS cells after a 1-h incubation at 37 °C with 10 μM compound **25**, followed by heating to 39°C-72 °C, with 3 °C increments. D. Protein levels of Nrf2 and its transcriptional target HO-1 following a 6-h incubation of Hepa1c1c7 cells with compound **25**. E. NQO1 specific enzyme activity in Hepa1c1c7 cells following a 48-h incubation of Hepa1c1c7 cells with compound **25**. F-J. mRNA levels for the Nrf2 target genes (NQO1, HO-1, GstP, Gclm, Gclc) following a 16-h incubation of Hepa1c1c7 cells with compound **25**.

The compounds were also evaluated for their ability to increase the expression of the Nrf2 target gene NQO1 in Hepa1c1c7 mouse hepatocyte cell lines. The compounds with the greatest fold induction at a concentration of 10 μM were the reference compound **2** (2.34-fold), the tetrazole compounds **13** (1.94-fold) and **25** (2.01-fold), along with the amide/acid **23** (1.90-fold). These were the compounds with nanomolar activity in the FP assay with the exception of **4** (0.94-fold NQO1 induction). Compounds **2** and **25** were also evaluated in an Nrf2 luciferase reporter assay in MCF7 cells [45]. Both compounds yielded similar activities at a concentration of 10 μM (**2** 1.39-fold induction of luciferase activity, **25** 1.48-fold), with peak induction measured at the highest test concentration of 100 μM (10.95-fold and 5.66-fold respectively) (Fig. 2B). Target engagement by compound **25** in the cellular environment was confirmed by the cellular thermal shift assay (CETSA) (Fig. 2C). Compound **25** increased the protein levels of Nrf2 and its transcriptional target heme oxygenase 1 (HO-1) (Fig. 2D), the specific enzyme activity of NQO1 (Fig. 2E), as well as the mRNA levels for a range of Nrf2 target genes (NQO1, HO-1, GstP, Gclm, Gclc) in Hepa1c1c7 cells (Fig. 2F–J). As previously reported, Keap1 binders in this class often have cellular activity at concentrations much higher than their Keap1 Kelch domain binding affinities. This is consistent with relatively poor cell penetration.

### 3.3 Further evaluation of acid/tetrazole compounds

Compounds **2**, **13** and **25** were further evaluated using isothermal titration calorimetry (ITC) and microscale thermophoresis (MST) to determine their dissociation constants for the Keap1 Kelch domain (Fig. 2A). The ITC data indicated that **2**, **13**, and **25** had a K_d_s of 39.9 ± 17.6 nM [23], 75.6 ± 33.9 nM and <1 nM respectively (Fig. S3). The corresponding *K*_d_ values measured using MST were 41.1 ± 5.8 nM, 17.1 ± 2.6 nM and 24.3 ± 4.7 nM for **2**, **13** and **25** respectively. The values for the MST dissociation constants are close to the limit of sensitivity for the assay, reducing the Keap1 protein concentration used in the assay from 10 nM to 1 nM gave a *K*_d_ value of 2.1 ± 0.5 nM for **25**. The data indicate that the asymmetric compound **25** has a high affinity interaction with the Keap1 Kelch domain (a *K*_d_ value ≤ ∼1 nM, the lower limit of detection for our measurements). The *bis*-acid **2** and *bis*-tetrazole **13** have higher *K*_d_ values in the low nanomolar range. Thus **25** had a higher binding affinity for Keap1 than would be expected for combining the acid and tetrazole substituents, a situation distinct from the corresponding acid amide compounds where the asymmetric analogue **23** had a binding affinity between that of **2** and the *bis*-amide **4**.

### 3.4 X-ray crystallography

**Fig. 3:**
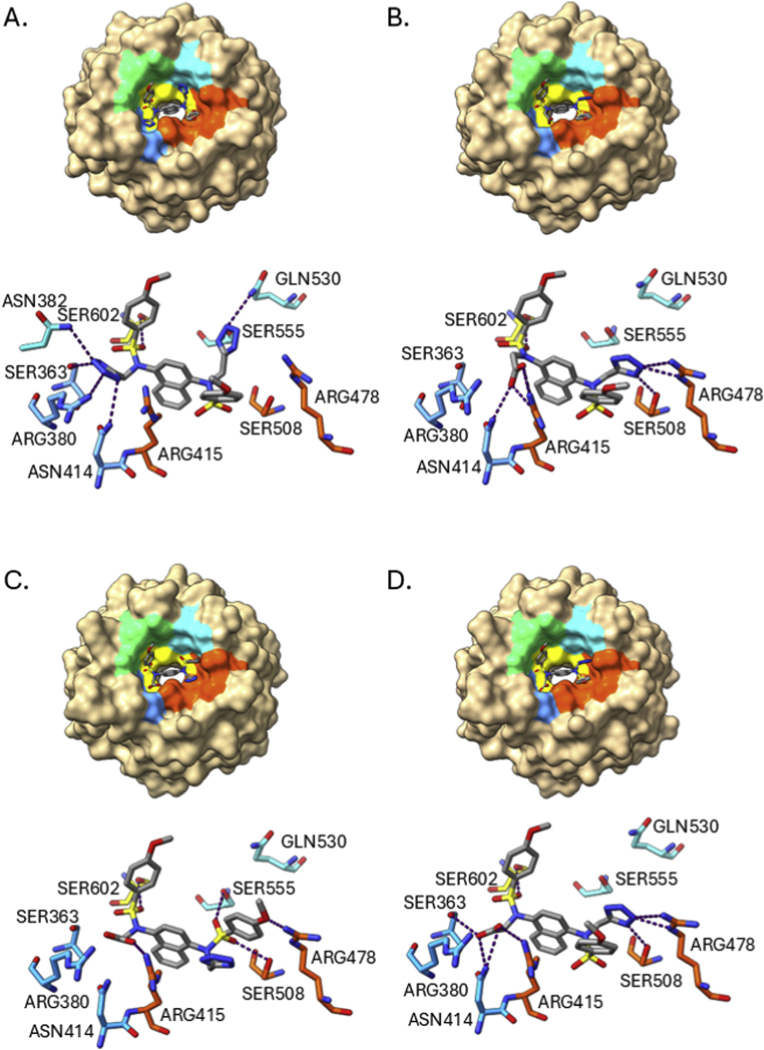
Bound conformations of compounds with the Keap1 Kelch domain. **13** (A.); **25**-trans (soaked in to Keap1 crystals at 4 °C) (B.); the two forms of **25** soaked in to Keap1 crystals at 18 °C, the *cis*-form (C.) and the *trans*-form (D.). Protein is shown as a surface (top) and stick (bottom) with sub-pockets P1 (orange, residues 415, 461, 462, 478, 483 & 508), P2 (blue, 363, 380, 381 & 414), P3 (yellow, 364, 509, 556, 571, 602 & 603), P4 (green, 334, 572 & 577) and P5 (cyan, 525, 530 & 555); compounds are shown in stick representation. Images were created using UCSF ChimeraX [9]. Hydrogen bonds were assigned using the ChimeraX H-Bonds tool and the default parameters and are shown as purple dotted lines.

Next, we investigated how the two tetrazole-containing compounds **13** and **25** bound to the Keap1 Kelch domain using X-ray crystallography. The *bis*-tetrazole **13** bound to Keap1, resolved at a resolution of 2.6 Å (PDB entry 9R1I), occupied an unexpected conformation with the tetrazoles (and sulfonamide substituents) adopting a *trans*-relationship about the central naphthalene ring (Fig. 3). This was unexpected because the previously reported crystal structure of the *bis*-amide compound **4** (PBD 4XMB) [18], the mono acid compound **5** (PBD 6V6Z) and the acid/trifluoromethyl isoquinoline **6** (PDB entry 6UF0) [21] have a *cis*-orientation of the sulfonamide substituents relative to the central bicyclic ring system. Compound **13** is positioned with a sulfonamide substituent subpocket P4 adjacent to the aromatic residues TYR334, TYR572, and PHE577 and a tetrazole in subpocket P2, forming polar interactions with SER363, ARG380, ASN382, and ASN414 (Fig. 3A). These latter interactions are observed in several crystal structures of carboxylic acids and amides bound to Keap1 (e.g. PDB entries 6V6Z, 6UF0, and 4XMB). In contrast the second tetrazole of **13** occupies the P5 subpocket in the vicinity of TYR525 and SER555 and the remaining sulfonamide occupies the P1 subpocket with the phenyl ring placed close to PHE478. In this complex the ARG415 guanidine is not parallel to the naphthalene ring system (as observed in PDB entry 4IQK) due to crowding from the adjacent tetrazole and sulfonamide substituents (Fig. 3A).

When the crystals of Keap1 were soaked with **25** at a low temperature (4 °C, PDB entry 9R1C) we observed a *trans*-type orientation of the molecule within the binding pocket similar to that of **13** (Fig. 3B). For **25**, the carboxylic acid occupying the P2 subpocket as expected and the opposing tetrazole at the boundary between the P1 and P5 subpockets. The tetrazole was in a pi-pi stacking arrangement with the adjacent phenylsulfonamide and positioned to form polar interactions with ARG483 from subpocket P1. Interestingly when the Keap1 crystals were soaked at a higher temperature (18 °C) the observed electron density suggested that **25** could be bound in either the cis or trans orientations (PDB entry 9R1Z) (Fig. 3C and D). The *trans*-conformation was largely identical to that observed for the 4 °C crystallisation, however the *cis*-form had the tetrazole in the P1 subpocket adjacent to ARG415 and PHE478. The apparent dual and temperature-sensitive binding modes for **25** and its high binding affinity to the Keap1 Kelch domain prompted us to evaluate the proportions of cis and trans species in solution using NMR and the predicted binding energies of each form using molecular dynamics simulations.

### 3.5 NMR

**Fig. 4:**
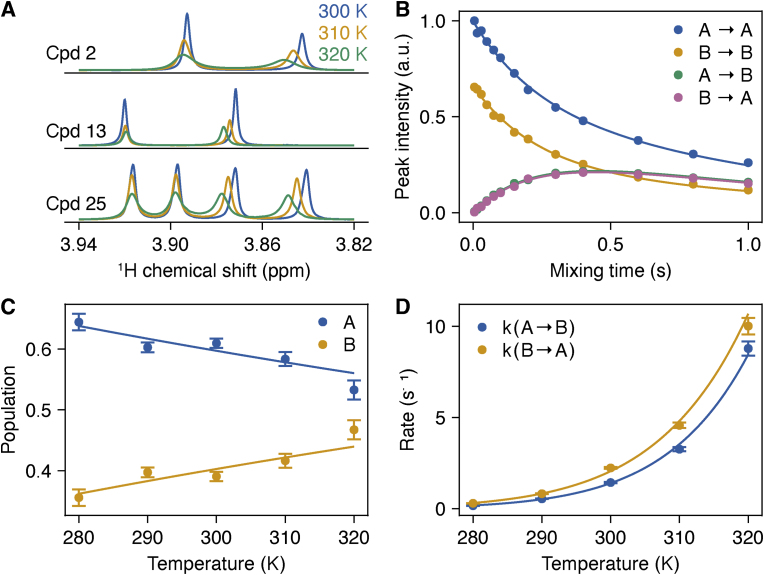
A. Variable temperature 1D ^1^H NMR spectra showing methoxy singlet resonances of **2**, **13** and **25** (d_6_-DMSO, 600 MHz). A and B state resonances have been labelled according to EXSY measurements (Fig. S2); B. Methoxy cross-peak intensities from ^1^H EXSY measurements of **25** as a function of the mixing time (100 % D_2_O, 300 K, 700 MHz). Data are fitted to determine the *R*_1_ relaxation rates of A and B (0.936 ± 0.034 s^−1^ and 0.842 ± 0.056 s^−1^ respectively) and the exchange rates *k*_AB_ = 1.432 ± 0.033 s^−1^, *k*_BA_ = 2.233 ± 0.049 s^−1^; C. Relative populations of A and B vs temperature, determined from EXSY measurements of **25** in D_2_O and fitted to the van ’t Hoff equation to determine ΔH_AB_ = 6.0 ± 1.3 kJ mol^−1^ and ΔS_AB_ = 16.8 ± 4.4 J K^−1^ mol^−1^; D. Interconversion rates of **25** in D_2_O determined from EXSY measurements and fitted to the Eyring equation to determine activation parameters ΔH^‡^_AB_ = 69.3 ± 0.9 kJ mol^−1^ and ΔS^‡^_AB_ = −11.3 ± 3.0 J K^−1^ mol^−1^.

The NMR spectra of compounds **2**, **13** and **25** showed doubling of the proton signals associated with the 4-methoxyphenylsulfonamide, N-alkyl substituents and adjacent naphthalene protons. This indicated that each compound exists as a pair of interconvertible rotamers in solution in an approximately 50:50 ratio with the sulfonamides either on the same face of the naphthalene ring (cis) or on opposite sides (trans) when viewed down the plane of the naphthalene bicycle. The phenylsulfonamide 4-methoxy resonances are well-resolved singlets that can be used to quantify the ratios of shielded (A) to deshielded (B) signals for each compound at three temperatures (300 K, 310 K and 320 K) (Fig. 4, Table S2). The compounds **2**, **13** and **25** were studied in DMSO solution and **25** was also evaluated in a D_2_O PBS buffer solution (pH∗ ∼7.4). Line shape analysis [40] was used to estimate the exchange rates between the A and B states for each compound at each temperature (Table S3, Fig. S1 and S2). The interconversion timescales were in the range 0.1–5 s. The interconversion rates for **25** in aqueous buffer were also determined using EXSY NMR data (Fig. 4B-D, Table S4). This gave values for the rate constants that were in good agreement with the line shape analysis data (1.432 vs. 1.432 s^−1^ and 2.205 vs. 2.233 s^−1^ for the A-B and B-A transitions respectively at 300 K). Using these data, we calculated the reaction and activation enthalpies (ΔH_AB_ and ΔH^‡^_AB_) for each transition using the van't Hoff and Eyring equations (Table S5).

The data indicate that the A to B transition in DMSO solution has a higher ΔH^‡^_AB_ for the *bis*-acid compound **2** and a lower barrier for the *bis*-tetrazole **13** (66.0 ± 2.5 kJ mol^−1^ vs. 43.6 ± 9.6 kJ mol^−1^ respectively). The asymmetrical acid/tetrazole **25** has a ΔH^‡^_AB_ value closer to that of the *bis*-tetrazole **13** (48.0 ± 13.0 kJ mol^−1^) (Table S5). The ΔH_AB_ values from the A:B equilibria followed a similar trend. The largest difference was measured for the *bis*-acid **2** (−2.66 ± 0.25 kJ mol^−1^) and the smallest for the *bis*-tetrazole **13** (−1.65 ± 0.17 kJ mol^−1^) with similar values observed for the asymmetric **25** (−1.72 ± 0.05 kJ mol^−1^). Compound **25** was also evaluated in D_2_O buffer in which it is anticipated that the carboxylic acid and/or tetrazole will be in their deprotonated forms. For this aqueous system we suggest that lower energy state B is likely to be the *trans*-form because the large sulfonamide substituents and the negatively charged acid and tetrazole are the maximum distance apart. There is also a larger ΔH^‡^_AB_ (69.3 ± 0.9 kJ mol^−1^) associated with moving to the A state (the proposed cis form) in which the negatively charged groups are closer together. The B state was more populated at 300 K with the difference reducing as the temperature increased from 300 K to 320 K (Table S4). The enthalpy difference between the states was also larger (6.0 ± 1.3 kJ mol^−1^ in D_2_O buffer vs. −1.723 ± 0.054 kJ mol^−1^ in DMSO). This energy difference suggests that the ratio of the A(cis):B(trans) states would be 1.6:1 at the 18 °C (291 K) crystallisation temperature, but 1.8:1 at the 4 °C (277 K) crystallisation temperature.

### 3.6 Molecular dynamics

**Fig. 5:**
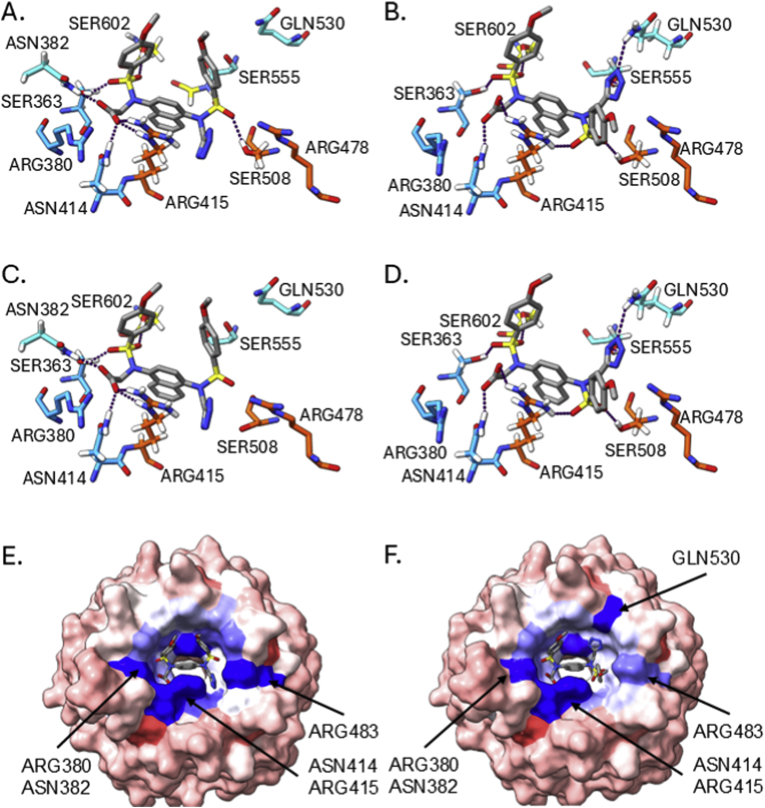
Time averaged structures from molecular dynamics. Compound **25** in its cis (A.) and trans (B.) bound forms simulated at 277 K and the cis (C.) and trans (D.) forms simulated at 300 K. Hydrogen bonds were assigned using the ChimeraX H-Bonds tool and the default parameters and are shown as purple dotted lines, hydrogens of interacting residues are shown in white. Compound **25** is shown as a grey stick model, the protein residues are coloured by subpocket (see Fig. 1, Fig. 3); MMGBSA energy decomposition of the interaction energies for the cis (E.) and trans (F.) forms of compound **25**, the surface colours correspond to favourable (blue), neutral (white) and unfavourable (red) contributions to the binding energies. Images were created using UCSF ChimeraX [9].

To gain further insights into the X-ray crystallography and NMR data we ran a series of molecular dynamics (MD) simulations using AMBER software and forcefields of **25** bound to the Keap1 Kelch domain in its cis and trans conformations at both 277 K (4 °C) and 300 K (27 °C) (3 × 1 μs per system). Analysis of the simulations showed that the protein and bound **25** were stable (RMSD <2 Å, Fig. S4). The time averaged structures from the simulations showed a high degree of similarity to the crystallised complexes (trans RMSD 0.492 Å; cis RMSD 0.513 Å at 300 K) and relatively limited mobility of **25** within the binding pocket (Fig. 5). MMGBSA energy calculations on the trajectories showed that the *cis*-conformation has a more favourable calculated interaction energy than the *trans*-form with Keap1 at both temperatures (cis −432.88 and −419.40 kJ mol^−1^ compared to trans −370.54 and −371.58 kcal/mol at 277 K and 300 K respectively) (Table 2, Fig. S5). This is consistent with the *cis*-form of the ligand forming a greater number of polar interactions with Keap1. There is relatively little temperature dependence on the binding energies for each system, although the difference between the cis and trans states is smaller at 300 K (−47.82 kJ mol^−1^ at 300 K cf. −62.34 kJ mol^−1^ at 277 K). The bound ligand energy when complexed with Keap1 was more negative for the *cis*-conformation (−419.40 kJ mol^−1^) than the trans (−371.58 kJ mol^−1^) at 300 K. This is in contrast to the free ligands simulated in solution at 300 K where the total energies from the MD indicate that the *trans*-form has a marginally lower mean energy (−707.01 ± 25.23 kJ mol^−1^) than the cis (−699.06 ± 26.74 kJ mol^−1^). The *cis*-form of the compound may be more favoured energetically in the binding pocket due to screening of the adjacent carboxylate and tetrazole anions by the arginine residues ARG380, 415 and 483 (Fig. 5A–D). Decomposition of the binding energies shows that these residues contribute significantly to the interaction with the compound in both cases, although ARG483 has fewer interactions with the *trans*-form of the compound because it is further away from the tetrazole (Fig. 5E and F).

**Table 2:** Compound **25** MM-GBSA energies calculated from MD trajectories.

	Calculated energy^a^ (KJ/mol)			
	*trans*-conformation		*cis*-conformation	
	277 K	300 K	277 K	300 K
Complex	−32,894.52 ± 14.93	−31,833.84 ± 30.84	−32,965.82 ± 44.77	−31,892.21 ± 22.64
Protein	−31,828.40 ± 29.79	−30,780.22 ± 26.07	−31,824.09 ± 35.65	−30,778.97 ± 22.13
Ligand	−695.59 ± 1.17	−682.08 ± 1.21	−708.85 ± 1.76	−693.87 ± 0.13
Difference	−370.54 ± 32.01	−371.58 ± 5.15	−432.88 ± 9.04	−419.40 ± 13.93

a Mean values (± standard deviation) from three independent 1 μs simulations at either 277 K (4 °C) or 300 K (27 °C). Mean simulation RMSD values: *trans*-conformation 1.512 ± 0.111 Å (277 K), 1.622 ± 0.095 Å (300 K); *cis*-conformation 1.469 ± 0.101 Å (277 K), 1.628 ± 0.105 Å (300 K).

## 4 Conclusions

Compound **25** has a high binding affinity for Keap1 (*K*_d_ < 1 nM) and is therefore amongst the tightest binders described to date. It inhibits the Keap1-Nrf2 protein-protein interaction and induces the expression of Nrf2 target genes, however this is observed at micromolar concentrations, probably due to poor cell permeability. It is not possible to be certain whether the cis or *trans*-form of **25** has a higher affinity for Keap1. NMR evaluation indicates that both states are occupied in solution and the interconversion between the states is relatively fast at the crystallisation temperatures suggesting that this plays little role in biasing the bound form of the compound. The *trans*-form is predicted to be the lower energy state in solution, with the charged groups on opposite faces of the molecule and the bulky sulfonamide groups far apart. The *cis*-form is predicted to bind more tightly due to a more extensive network of polar interactions between the carboxylic acids, tetrazoles and ARG 380, 415, and 483. The data are consistent with the P2 subpocket of Keap1 invariably accommodating a polar group (carboxylate, amide, tetrazole) and the P1 and P5 sub-pockets having less strict requirements. The structural data support our previous observations that molecules with adjacent planar aromatic groups may stack in order to occupy a large and relatively open binding pocket such as that present in the Kelch domain of Keap1. Together with our previous study on phenyl *bis*-sulfonamides [23], this work provides further insights into the diversity of binding modes for this chemotype of Keap1 inhibitor and informs their future development. It also indicates that caution may be required when making assumptions about the binding modes of closely related compounds in SAR studies.

## Accession codes

PDB ID of New Crystal (X-ray) Structures: Atomic coordinates and experimental data for compounds **13** and **25** (soaked at 4 °C or 18 °C) bound to the Keap1 Kelch domain are available: PDB entry 9R1I, 9R1C and 9R1Z respectively.

## CRediT authorship contribution statement

**Nikolaos D. Georgakopoulos:** Conceptualization, Investigation, Methodology. **Sandeep K. Talapatra:** Data curation, Investigation, Methodology, Writing – review & editing. **Sharadha Dayalan Naidu:** Investigation, Methodology. **Dina Dikovskaya:** Investigation, Methodology. **Maureen Higgins:** Investigation, Methodology. **Jemma Gatliff:** Investigation, Methodology. **Roxani Nikoloudaki:** Investigation, Methodology. **Marjolein Schaap:** Investigation, Methodology. **Jasmine M. Walker:** Investigation, Methodology. **Christopher Wardby:** Investigation, Methodology, Writing – original draft. **Albena T. Dinkova-Kostova:** Formal analysis, Funding acquisition, Supervision, Writing – original draft, Writing – review & editing. **Sarah Harris:** Investigation, Writing – original draft, Writing – review & editing. **Frank Kozielski:** Supervision, Writing – original draft, Writing – review & editing. **Geoff Wells:** Conceptualization, Funding acquisition, Project administration, Supervision, Writing – original draft, Writing – review & editing.

## Declaration of competing interest

The authors declare that they have no known competing financial interests or personal relationships that could have appeared to influence the work reported in this paper.

## Data Availability

Data will be made available on request.

## Supporting Information

X-ray crystallography statistics, molecular dynamics summary data, ITC data, and analytical data for new tested compounds.

## Abbreviations Used:

ARE: antioxidant response element
CETSA: cellular thermal shift assay
DPBS: Dulbecco's phosphate buffered saline
DSF: differential scanning fluorimetry
FP: fluorescence polarisation
GstP: glutathione S-transferase P
Gclc: glutamate-cysteine ligase catalytic subunit
Gclm: glutamate-cysteine ligase modulatory subunit
HEPES: 4-(2-hydroxyethyl)-1-piperazineethanesulfonic acid
Hmox1: heme oxygenase 1
ITC: isothermal titration calorimetry
Keap1: Kelch-like ECH-associated protein 1
MD: molecular dynamics
MMGBSA: molecular mechanics-generalized Born surface area
MOPS: 3-(N-morpholino)propanesulfonic acid
MST: microscale thermophoresis
MTT: 3-(4,5-dimethylthiazol-2-yl)-2, 5-diphenyltetrazolium bromide
Neh2: Nrf2−ECH homology 2 domain
NQO1: NAD(P)H quinone oxidoreductase 1 or NAD(P)H quinone dehydrogenase 1
Nrf2: nuclear factor erythroid 2-related factor 2
PBS: phosphate buffered saline
PI: protein-protein interaction
RMSD: root mean square deviation
Ti: inflection temperature
ΔTi: change in inflection temperature
