# Supplementary material for: Tetrazole-containing naphthalene bis-sulfonamide Keap1-Nrf2 interaction inhibitors with unexpected binding modes
Source: Redox Biol. 2025 Nov 10;88:103924. doi: 10.1016/j.redox.2025.103924 (PMC12664080; doi:10.1016/j.redox.2025.103924)
Supplement: Multimedia component 1 [file mmc1.docx]

**SUPPLEMENTARY DATA**

**Tetrazole-containing naphthalene *bis*-sulfonamide Keap1-Nrf2 interaction inhibitors with unexpected binding modes**

Nikolaos D. Georgakopoulos^1^, Sandeep K. Talapatra^1^, Sharadha Dayalan Naidu^2^, Dina Dikovskaya^2,3^, Maureen Higgins^2^, Jemma Gatliff^1,4^, Roxani Nikoloudaki^1^, Marjolein Schaap^1^, Jasmine M. Walker^2^, Christopher Wardby^1^, Albena T. Dinkova-Kostova^2,5^, Sarah Harris^6^, Frank Kozielski^1^ and Geoff Wells^1^

^1^UCL School of Pharmacy, University College London, 29/39 Brunswick Square, London, WC1N 1AX, UK, ^2^Jacqui Wood Cancer Centre, Division of Cellular Medicine, University of Dundee School of Medicine, Dundee, DD1 9SY, Scotland, UK, ^3^ Peninsula Medical School, University of Plymouth, Plymouth, Devon PL4 8AA, UK, ^4^Keregen Therapeutics Ltd, Stevenage Bioscience Catalyst, Gunnels Wood Rd, Stevenage, SG1 2FX, UK, ^5^Department of Pharmacology and Molecular Sciences and Department of Medicine, Johns Hopkins University School of Medicine, Baltimore, MD 21205, USA, ^6^Department of Physics and Astronomy, Hicks Building, Hounsfield Rd, Broomhall, Sheffield S3 7RH, UK.

Corresponding author: Geoff Wells, E: g.wells@ucl.ac.uk; T: 020 3987 2819

**X-ray Crystallography Parameters**

**Table S1.** Data collection, data processing, and model refinement statistics for Keap1-Compound **13** and Keap1-Compound **25** complexes. Data in parenthesis correspond to the highest resolution shell.

| Data collection and Refinement Statistics | Keap1-Compound **13** | Keap1-Compound **25** (18°C) | Keap1-Compound **25** (4°C) |
| --- | --- | --- | --- |
| Wavelength [Å] | 0.9766 | 0.9800 | 0.9660 |
| Resolution range [Å] | 40.82 - 2.592 (2.685 - 2.592) | 32.78 - 1.5 (1.554 -1.5) | 46.75 - 1.693 (1.754 - 1.693) |
| Space group | C 1 2 1 | | |
| Unit cell parameters [Å;°] | a=136.277, b=75.468, c=121.77, 𝝰= 𝝲= 90, 𝝱=159.137 | a=126.34, b=75.461, c=48.63, 𝝰= 𝝲= 90, 𝝱=106.36 | a=126.419, b=75.58, c=48.680, 𝝰= 𝝲= 90, 𝝱=106.19 |
| Molecules per asymmetric unit | 1 | | |
| Total reflections | 40890 (6110) | 136691 (13451) | 198543 (25291) |
| Unique reflections | 13516 (1284) | 69847 (6945) | 48908 (4843) |
| Multiplicity | 3.0 (3.1) | 4.1 (3.9) | 4.1 (3.6) |
| Completeness [%] | 98.10 (93.42) | 99.59 (99.21) | 99.75 (99.90) |
| Mean I/sigma(I) | 5.5 (2.0) | 8.7 (1.9) | 6.9 (2.4) |
| Wilson B-factor | 36.32 | 16.66 | 14.44 |
| R-means [%] | 22.4 (61.0) | 10.6 (99.0) | 14.5 (58.5) |
| Rpim [%] | 12.5 (59.7) | 5.2 (55.7) | 7.0 (30.1) |
| CC_1/2_ [%] | 97.0 (30.7) | 99.7 (41.3) | 99.4 (43.7) |
| Reflections used in refinement | 13505 (1278) | 69767 (6915) | 48887 (4843) |
| R_cryst_/R_free_ [%] | 18.6 (30.6) / 23.7 (36.4) | 16.6 (27.0) / 18.7 (30.1) | 18.2 (30.5) / 21.5 (34.0) |
| Total no. of non-hydrogen atoms (protein) | 2293 | 2719 | 2692 |
| No. of protein/ligand/solvent atoms | 2192 / 46 / 55 | 2227 / 48 / 444 | 2227 / 44 / 421 |
| RMSD bond length, bond angles [Å;°] | 0.012 / 1.26 | 0.012 / 1.20 | 0-012 / 1.23 |
| Ramachandran  Favored / allowed / outliers / rotamer outliers [%] | 95.4 / 4.6 / 0 / 1.7 | 97.9 / 2.1 / 0.9 / 0.4 | 97.9 / 2.1 / 0.0 / 0.9 |
| Clashscore | 8.1 | 3.0 | 3.6 |
| Average B-factor / protein / ligands / solvent | 46.8 / 46.3 / 72.5 / 44.5 | 23.0 / 20.5 / 28.8 / 34.9 | 19.6 / 17.3 / 24.0 / 31.1 |

**NMR Parameters and Spectra**

**Table S2.** Populations of cis and trans species in solution estimated by NMR lineshape analysis at different temperatures.

| Compound | Occupancy of the more shielded state (A)^a^ | | |
| --- | --- | --- | --- |
|  | 300 K | 310 K | 320 K |
| **2** | 0.426 ±0.001 | 0.436 ±0.001 | 0.443 ±0.001 |
| **13** | 0.542 ±0.003 | 0.548 ±0.001 | 0.552 ±0.001 |
| **25** | 0.486 ±0.003 | 0.491 ±0.001 | 0.496 ±0.0005 |
| **25** (D_2_O buffer)^b^ | 0.606 ±0.003 | 0.586 ±0.001 | 0.567 ±0.001 |

Notes: a. The compound form with a high-field CH_3_ resonance from the sulfonamide 4-methoxy substituent. b. D_2_O PBS buffer (pH* ~7.4).

**Table S3.** Rate constants for interconversions between cis and trans forms estimated by lineshape analysis.

| Compound | Rate constants for A:B interconversion at different temperatures | | | | | |
| --- | --- | --- | --- | --- | --- | --- |
|  | 300 K | | 310 K | | 320 K | |
|  | k_A→B_ (s^-1^) | k_B→A_ (s^-1^) | k_A→B_ (s^-1^) | k_B→A_ (s^-1^) | k_A→B_ (s^-1^) | k_B→A_ (s^-1^) |
| **2** | 3.175 ±0.005 | 2.360 ±0.004 | 8.456 ±0.024 | 6.541 ±0.018 | 18.949 ±0.04 | 15.054 ±0.03 |
| **13** | 1.063 ±0.008 | 1.257 ±0.010 | 1.459 ±0.006 | 1.770 ±0.007 | 2.927 ±0.010 | 3.608 ±0.012 |
| **25** | 3.059 ±0.029 | 2.887 ±0.027 | 3.982 ±0.007 | 3.838 ±0.007 | 8.749 ±0.012 | 8.621 ±0.012 |
| **25** (D_2_O buffer)^b^ | 1.432 ±0.015^c^ | 2.205 ±0.022^c^ | 3.488 ±0.014^c^ | 4.933 ±0.02^c^ | 8.912 ±0.024^c^ | 11.65 ±0.034^c^ |

Notes: a. ‘A’ is defined to be the compound form with a more shielded CH_3_ resonance from the sulfonamide 4-methoxy substituent. b. D_2_O PBS buffer (pH* ~7.4); c. Rate constants for **25** (D_2_O buffer) using EXSY measurements provided in Table S4.

**Table S4.** Rate constants for interconversions of compound **25** between cis and trans forms in D_2_O PBS buffer determined by exchange spectroscopy (EXSY).

| Temperature (K) | k_A→B_ (s^-1^) | k_B→A_ (s^-1^) |
| --- | --- | --- |
| 280 | 0.1633 ± 0.0072 | 0.296 ± 0.012 |
| 290 | 0.541 ± 0.013 | 0.821 ± 0.019 |
| 300 | 1.432 ± 0.033 | 2.233 ± 0.049 |
| 310 | 3.26 ± 0.11 | 4.57 ± 0.15 |
| 320 | 8.78 ± 0.39 | 10.01 ± 0.45 |

**Table S5.** Enthalpies and entropies of activation and reaction for interconversion of cis and trans rotamers determined by NMR lineshape analysis.

| Compound | ΔH^‡^_AB_ (KJ mol^-1^) | ΔH_AB_ (KJ mol^-1^) | ΔS^‡^_AB_ (KJ mol^-1^) | ΔS_AB_ (KJ mol^-1^) |
| --- | --- | --- | --- | --- |
| **2** | 66.0 ± 2.5 | -2.66 ± 0.25 | -14.6 ± 7.9 | -6.42 ± 0.8 |
| **13** | 43.6 ± 9.6 | -1.65 ± 0.17 | -100 ± 30 | -6.89 ± 0.55 |
| **25** | 48.0 ± 13.0 | -1.723 ± 0.054 | -79 ± 41 | -5.26 ± 0.17 |
| **25** (D_2_O)^a^ | 73.0 ± 2.3 | 6.532 ± 0.022 | 0.65 ± 7.2 | 18.19 ± 0.07 |
| **25** (D_2_O)^a,b^ | 69.3 ± 0.9 | 6.0 ± 1.3 | -11.3 ± 3.0 | 16.8 ± 4.4 |

Notes : a. D_2_O PBS buffer, pH* ~7.4. b. Determined by exchange spectroscopy (EXSY).


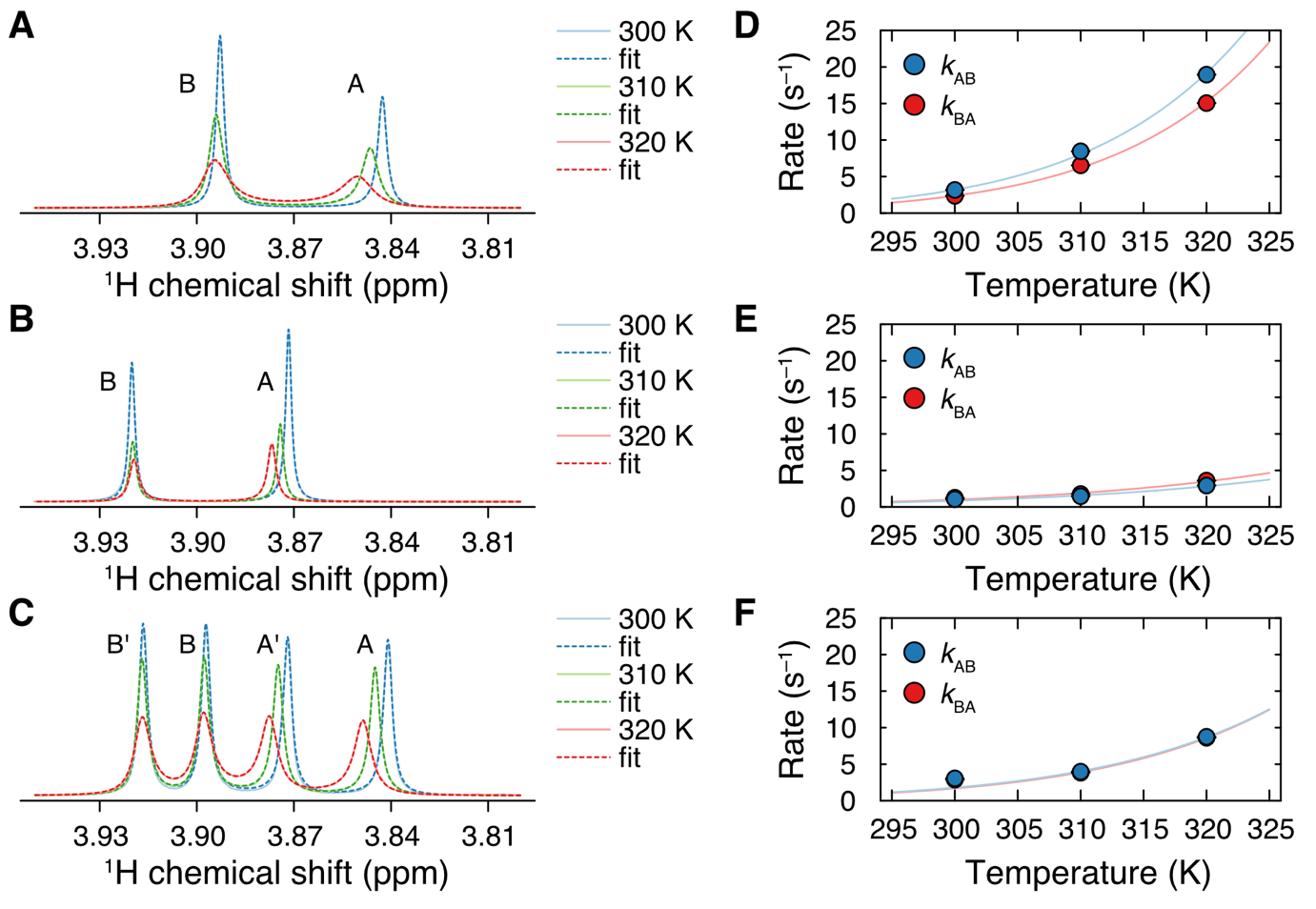


**Figure S1.** 1D ^1^H NMR spectra of (A) compound **2**, (B) compound **13**, and (C) compound **25** in DMSO vs temperature as indicated, and lineshape fits assuming an exchange-free relaxation rate of 3 s^-1^. (D-F) Estimated exchange rate constants from lineshape analysis vs temperature, (D) compound **2**, (E) compound **13**, and (F) compound **25**, fitted to Eyring equations (Table S3).


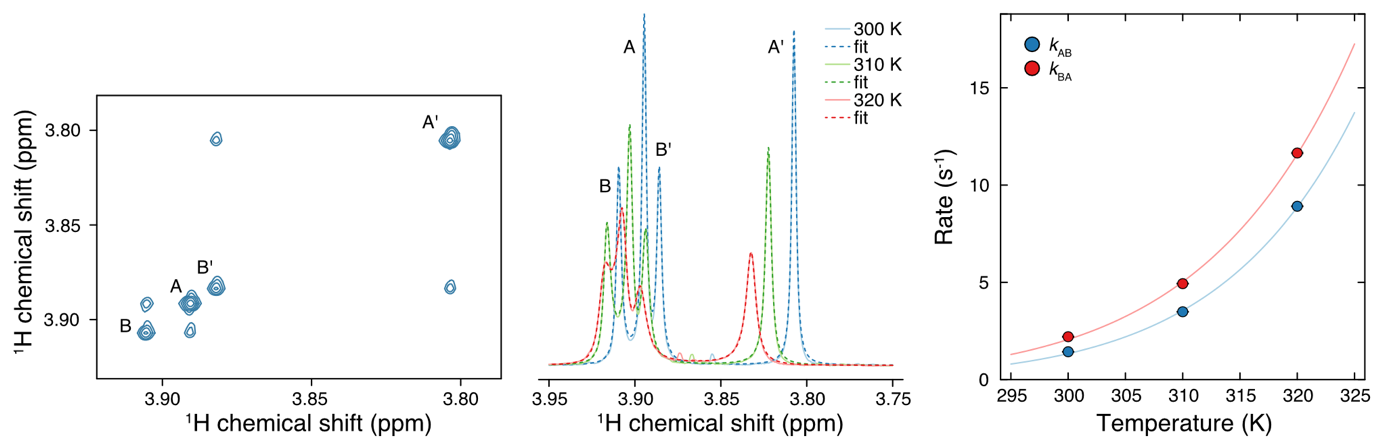


**Figure S2.** EXSY and 1D NMR lineshape analysis of compound **25** in D_2_O. (A) ^1^H EXSY spectrum of compound **25** highlighting methoxy resonances, acquired with a 200 ms mixing time at 300 K. (B) 1D ^1^H NMR spectra of compound **25** in D_2_O vs temperature, and lineshape fits assuming an exchange-free relaxation rate of 4 s^-1^. (C) Estimated exchange rate constants from lineshape analysis vs temperature, fitted to Eyring equations (Table S4).

**Compound 13**


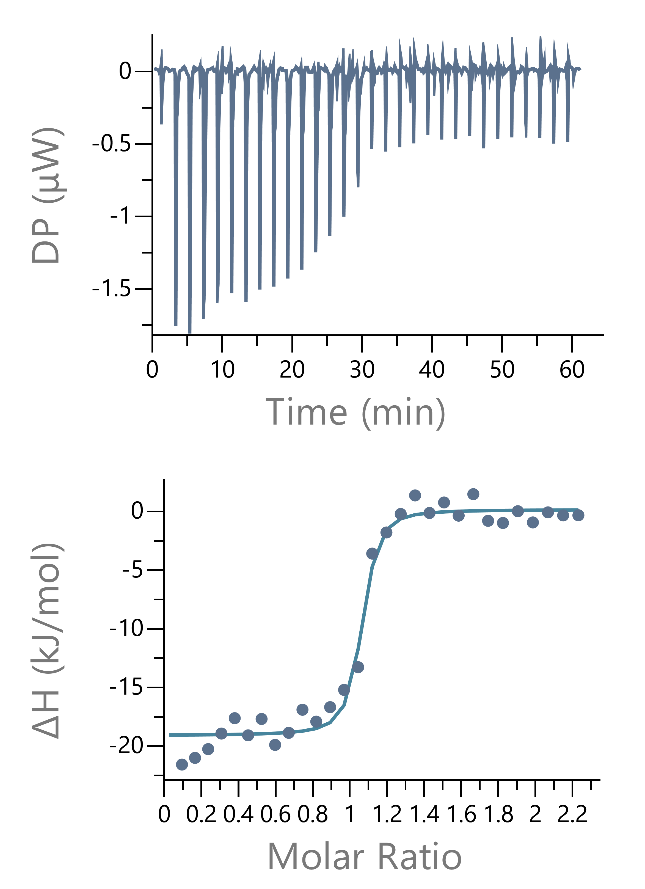


**Compound 25**

**
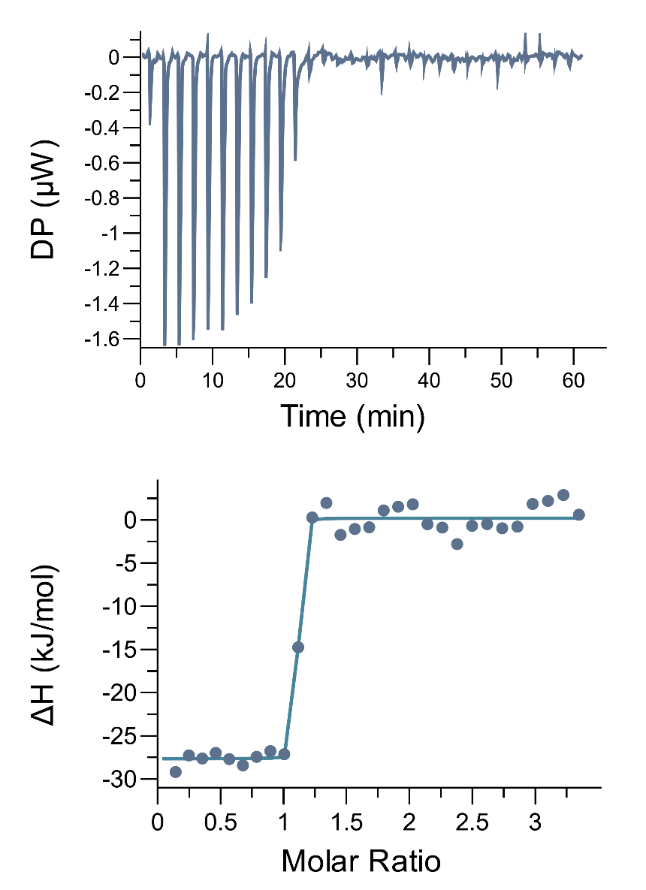
**

**Figure S3. ITC Keap1 interaction data for new tested compounds**

**Molecular Dynamics**

**Figure S4.** RMSD vs time plots for each of the Keap1 Kelch domain simulations with compound **25** bound in its cis or trans-forms. Each simulation was run at 300 K or 277 K and repeated three times (top row trans-**25** @ 300 K, second row trans-**25** @277 K, third row cis-**25** @300 K, forth row cis-**25** @277 K).

**Figure S5.** Energy difference (complex energy – protein energy – compound energy) vs time plots for each of the Keap1 Kelch domain simulations with compound **25** bound in its cis or trans-forms. Each simulation was run at 300 K or 277 K and repeated three times (top row trans-**25** @ 300 K, second row trans-**25** @277 K, third row cis-**25** @300 K, forth row cis-**25** @277 K).

**Analytical data for new tested compounds**

**HPLC**

Compound **12**

Compound **13**

Compound **17**

Compound **22**

Compound **23**

Compound **25**

**NMR spectra**
